# Isolation and Characterization of Novel Pueroside B Isomers and Other Bioactive Compounds from *Pueraria lobata* Roots: Structure Elucidation, *α*-Glucosidase, and *α*-Amylase Inhibition Studies

**DOI:** 10.3390/ijms25179602

**Published:** 2024-09-04

**Authors:** Wei Dai, Manqiu Lei, Qiuxiong Yin, Haijun Nan, Guoqiang Qian

**Affiliations:** 1 Teaching and Experimental Center, Guangdong Pharmaceutical University, Guangzhou 510006, China; 2 Comprehensive Experimental Teaching Center of Traditional Chinese Medicin, Yunfu Campus, Guangdong Pharmaceutical University, Yunfu 527500, China; 3 School of Chinese Materia Medica, Guangdong Pharmaceutical University, Guangzhou 510006, China; 4 School of Chinese Medicine, Guangdong Pharmaceutical University, Guangzhou 510006, China

**Keywords:** *Pueraria lobata*, chemical constituents, *α*-glucosidase inhibition, *α*-amylase inhibition, 4*R*-pueroside B, molecular docking

## Abstract

*Pueraria lobata* (Willd.) Ohwi is a traditional medicinal herb that has been extensively used in Chinese medicine for various therapeutic purposes. In this study, twelve chemical constituents were isolated from the roots of *P. lobata*, comprising three puerosides (compounds **1**–**3**), six alkaloids (compounds **4**–**9**), and three additional compounds (compounds **10**–**12**). Notably, compound **1** (4*R*-pueroside B) was identified as a novel compound. The structures of all compounds were elucidated using a range of spectroscopic techniques, including CD spectroscopy for the first-time determination of the absolute configurations of pueroside B isomers (compounds **1** and **2**). Enzyme inhibition assays revealed that, with the exception of compound **2**, all isolated compounds exhibited varying degrees of *α*-glucosidase and *α*-amylase inhibitory activity. Remarkably, compound **12** demonstrated IC_50_ values of 23.25 μM for *α*-glucosidase inhibition and 27.05 μM for *α*-amylase inhibition, which are superior to those of the positive control, acarbose (27.05 μM and 36.68 μM, respectively). Additionally, compound **11** exhibited inhibitory activity against *α*-glucosidase and *α*-amylase comparable to the positive control, acarbose. Molecular docking studies indicated that compound **12** interacts with the active sites of the enzymes via hydrogen bonds, van der Waals forces, and hydrophobic interactions, which likely contribute to their inhibitory effects. These findings suggest that the chemical constituents of *P. lobata* could be potential natural sources of *α*-amylase and *α*-glucosidase inhibitors, with compound **12** being particularly promising for further investigation.

## 1. Introduction

*Pueraria lobata* (Willd.) Ohwi, a perennial climbing plant belonging to the genus Pueraria in the Fabaceae family, is widely distributed in temperate and subtropical regions [1]. The underground part of *P. lobata*, commonly known as ‘ge-gen’, has a long history of use in traditional Chinese medicine, first recorded in the famous Chinese medical work ‘Shennong Ben Cao Jing’ [2]. It is known for its properties of releasing exterior, reducing fever, promoting fluid production to quench thirst, and stopping diarrhea by warming the middle energizer [3]. It is used to treat various ailments such as cardiovascular diseases, fever, headache, diabetes, alcohol poisoning, and diarrhea [4]. Besides its therapeutic uses, *P. lobata* is also widely employed in nutraceuticals and dietary supplements due to its rich medicinal and nutritional value, earning it the title of ‘Asian ginseng’ [5,6].

Recent in-depth research on *P. lobata* has led to the identification of various active components. According to literature reports, over 100 chemical constituents have been identified in *P. lobata*, including isoflavones, triterpenoids, coumarins, alkaloids, and more [5,7,8,9]. Isoflavones, such as puerarin, daidzin, daidzein, and kakkalide, are considered the main active components [10,11]. *P. lobata*, a key element in traditional Chinese medicine, has a long history of use in diabetes treatment. Modern scientific studies have further elucidated its antidiabetic mechanisms, which involve reducing insulin resistance, enhancing insulin secretion, inhibiting glucose absorption and reabsorption, and improving insulin sensitivity and metabolic processes [6]. For example, it has been demonstrated that *P. lobata* polysaccharides significantly improve metabolic profiles and insulin resistance in type 2 diabetic mice by modulating gut microbiota and metabolic pathways [12]. Additionally, the anti-inflammatory effects of *P. lobata* are primarily mediated through the regulation of arachidonic acid and glutathione metabolic pathways [13]. In terms of hepatoprotection, isoflavones and triterpene saponins in *P. lobata* play crucial roles in regulating oxidative stress, inflammatory responses, and lipid metabolism [5]. Furthermore, the anticancer potential of *P. lobata* has been explored, highlighting the molecular mechanisms through which daidzein exerts anticancer effects via multiple cell signaling pathways [14]. These findings provide a robust scientific foundation for the application of *P. lobata* in treating diabetes and its complications, offering new directions for future research and clinical practice.

Enzymes are crucial indicators for disease diagnosis and important targets in drug discovery [15]. *α*-Amylase and *α*-glucosidase are key enzymes that regulate postprandial blood glucose levels. By inhibiting the activity of these enzymes, the absorption of carbohydrates in the upper small intestine is reduced, thereby delaying glucose absorption and lowering postprandial blood glucose levels [16,17]. While numerous studies have documented the inhibitory effects of *P. lobata*’s active components on *α*-glucosidase, with isoflavones being the primary contributors to this activity [18,19,20], research on other bioactive components of the plant remains limited. This study aims to explore the effects of non-isoflavonoid components, such as pueraria glycosides, alkaloids, and triterpenoids, on *α*-amylase and *α*-glucosidase inhibition. Using a combination of advanced chromatographic techniques and spectroscopic methods, we aim to elucidate the structures of these compounds and evaluate their inhibitory effects on key enzymes involved in carbohydrate digestion, specifically *α*-glucosidase and *α*-amylase. Through this investigation, we seek to uncover new insights into the chemical diversity of *P. lobata* and its potential applications in diabetes therapy.

## 2. Results and Discussion

### 2.1. Isolation and Structural Identification of Chemical Constituents from P. lobata

**Figure 1:**
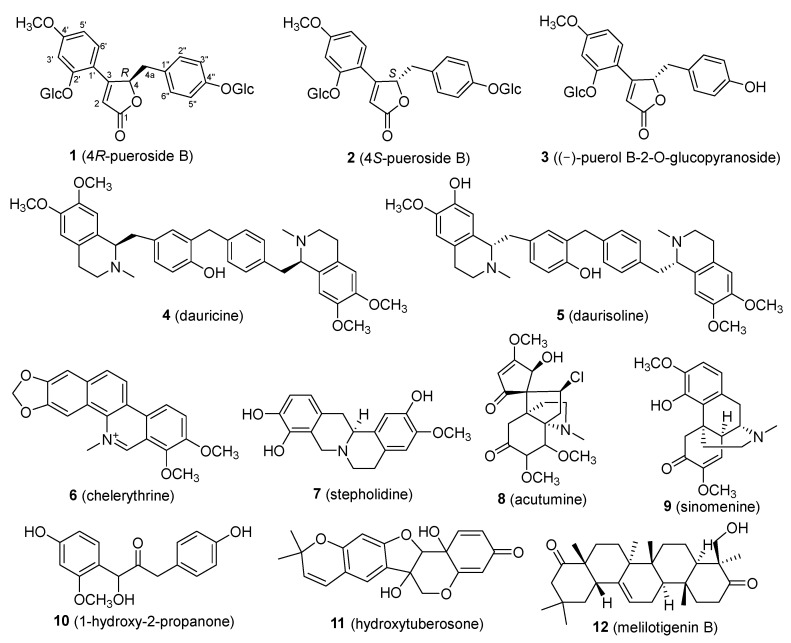
Structures of compounds **1**–**12**.

Through the investigation of natural products from the roots of *P. lobata*, a total of twelve compounds were isolated. These included three puerosides (compounds **1**–**3**), six alkaloids (compounds **4**–**9**), one phenolic compound (compound **10**), one flavonoid (compound **11**), and one triterpenoid (compound **12**). Compounds **1** and **2** are isomers, and their absolute configurations were determined for the first time as 4*R*-pueroside B and 4*S*-pueroside B, respectively, with compound **1** being a novel compound. The structures of the isolated compounds are shown in Figure 1. Notably, except for compounds **2** and **3**, all other compounds were isolated and identified from *P. lobata* for the first time. The structures of the known compounds (compounds **2**–**12**) were confirmed by comparing their spectral data with the literature values. The identified compounds include 4*S*-pueroside B (**2**) [21], (−)-puerol B-2-O-glucopyranoside (**3**) [22], dauricine (**4**) [23], daurisoline (**5**) [24], chelerythrine (**6**) [25], stepholidine (**7**) [26], acutumine (**8**) [27], sinomenine (**9**) [28], 1-hydroxy-2-propanone (**10**) [29], hydroxytuberosone (**11**) [30], and melilotigenin B (**12**) [31].

### 2.2. Isolation and Structural Elucidation of Compound ***1***

**Figure 2:**
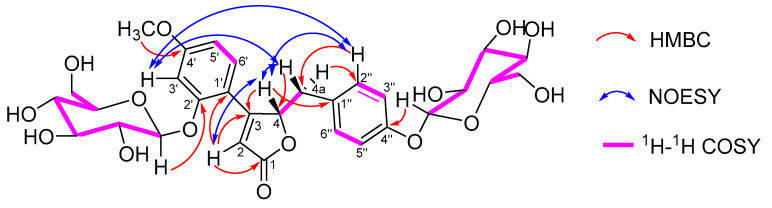
Key HMBC, COSY, and NOESY correlations of compound **1**.

Compound **1** was obtained as a white amorphous powder. The ESI-HR-MS gave an *m*/*z* value of 637.21307 [M + H]^+^, consistent with the molecular formula C_30_H_37_O_15_. The IR spectrum showed absorption bands corresponding to OH (3381 cm^−1^), ester carbonyl (1721 cm^−1^), and phenyl ring (1609, 1508 cm^−1^) moieties. The ^1^H NMR spectrum (Table 1) displayed a typical ABX system for a benzene ring at *δ*_H_ 7.47 (1H, d, *J* = 8.7 Hz, H-6′), *δ*_H_ 6.90 (1H, d, *J* = 2.4 Hz, H-3′), and *δ*_H_ 6.74 (1H, dd, *J* = 2.4, 8.7 Hz, H-5′). An AA′BB′ system for another benzene ring appeared at *δ*_H_ 6.94 (2H, d, *J* = 8.6 Hz, H-2″, 6″) and *δ*_H_ 6.87 (2H, d, *J* = 8.6 Hz, H-3″ 5″). Additionally, a methoxy group was observed at *δ*_H_ 3.84 (3H, s), an olefinic proton at *δ*_H_ 6.47 (1H, br s, H-2), an oxygenated methine proton at *δ*_H_ 6.02 (1H, m, H-4), CH_2_ protons at *δ*_H_ 2.71 (1H, dd, *J* = 14.7, 6.5 Hz, H-4a) and 3.15 (1H, m, H-4a), and two *β*-glucosyl units at *δ*_H_ 5.12 (1H, d, *J* = 7.6 Hz, glc-1‴) and 4.81 (1H, d, *J* = 7.7 Hz, glc-1⁗). The ^13^C NMR spectrum displayed 30 carbon signals, including one methoxy group at *δ*_C_ 55.5 (OCH_3_), two *β*-glucosyl units, and the remaining 17 carbons consistent with the core structure of pueroside, as confirmed by comparison with the literature data [21,22]. The ^1^H-^1^1H COSY NMR analysis further supported that this compound is an analogue of pueroside. The positions of the methoxy and *β*-glucosyl units were determined by HMBC correlation (Figure 2). The methoxy group at *δ*_H_ 3.84 (3H, s) showed a correlation with *δ*_C_ 162.6 (C-4′), confirming its position at C-4′. The *β*-glucosyl units at *δ*_H_ 5.12 (1H, d, *J* = 7.6 Hz, glc-1‴) and 4.81 (1H, d, *J* = 7.7 Hz, glc-1⁗) correlated with *δ*_C_ 157.1 (C-2′) and *δ*_C_ 156.2 (C-4″), respectively, confirming their positions at C-2′ and C-4″. Comparative analysis revealed that the planar structure of this compound was identical to pueroside B (compound **2**). Further analysis via liquid chromatography demonstrated the separation of these two compounds, with different retention times (Figure S11). Consequently, it was determined that this compound and pueroside B are isomers [21]. Examination of the core structure revealed circular dichroism (CD) at position 4, suggesting that the difference between these compounds arises from the configuration at this position. As in previous studies, NOESY correlations could not determine the configuration at position 4 (specific correlation signals shown in Figure 2).

**Figure 3:**
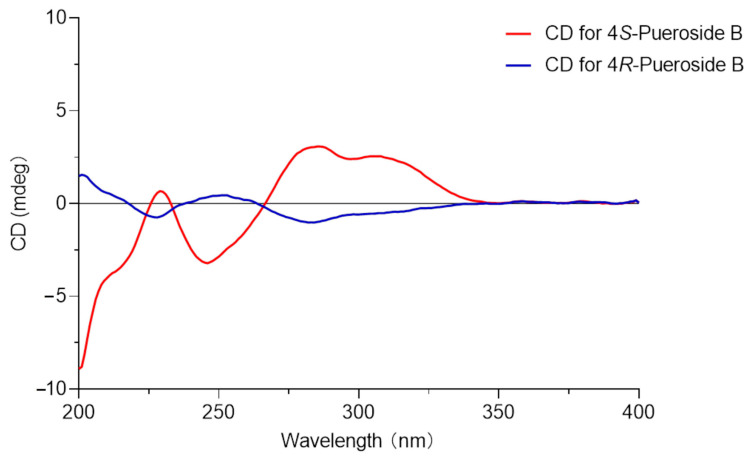
CD spectra of compounds **1** (4*R*-pueroside B) and **2** (4*S*-pueroside B).

To establish the absolute configuration of compounds **1** and **2**, CD spectral analysis was performed. Compound **1** exhibited a negative Cotton effect at 228 nm and 286 nm and a positive Cotton effect at 252 nm, whereas compound **2** showed the opposite, with a positive Cotton effect at 229 nm and 286 nm and a negative Cotton effect at 246 nm (Figure 3) [22,32]. Comparison of the CD spectra with literature data ultimately determined the absolute configurations of compounds **1** and **2** as 4*R*-pueroside B and 4*S*-pueroside B, respectively.

### 2.3. Mass Spectrometry Fragmentation Patterns of 4R-pueroside B and 4S-pueroside B

**Figure 4:**
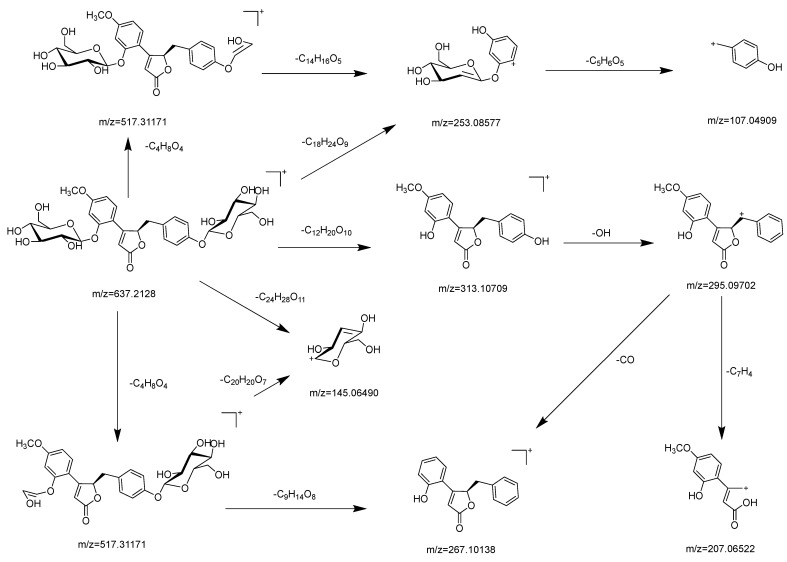
Probable mass spectrometric fragmentation pathway of pueroside B.

The study of the secondary mass spectrometry (MS/MS) fragmentation patterns of 4*R*-pueroside B and 4*S*-pueroside B revealed that these isomers exhibit highly similar fragmentation behaviors (MS/MS spectra shown in supporting information Figure S12). Consequently, the two isomers cannot be distinguished based on their MS/MS fragments alone. The specific fragmentation patterns are illustrated in Figure 4. Pueroside B primarily undergoes glycosidic bond cleavage, carbon–carbon bond cleavage within the core structure, or ring-opening to produce secondary fragments. The major fragmentation pathways include the complete glycosidic bond cleavage, forming the fragment at *m*/*z* 313.10709 ([M+H−C_12_H_20_O_10_]^+^). Further cleavage of the hydroxyl group on the benzene ring produces the fragment at *m*/*z* 295.09702 ([M+H−C_12_H_20_O_10_−OH]^+^), and the loss of the methoxy group yields the fragment at *m*/*z* 267.10138 ([M+H−C_12_H_20_O_10_−OH−CO]^+^). Alternatively, the five-membered ring opening of the fragment at *m*/*z* 295.09702 results in the formation of the fragment at *m*/*z* 207.06522 ([M+H−C_12_H_20_O_10_−OH−C_7_H_4_]^+^).

Additionally, the formation of the glycosyl fragment followed by the loss of one molecule of H_2_O produces the fragment at *m*/*z* 145.06490 ([M+H−C_24_H_28_O_11_]^+^). In the case of incomplete glycosidic bond cleavage, the fragment at *m*/*z* 517.31171 ([M+H−C_4_H_8_O_4_]^+^) is formed. Further cleavage of the carbon–carbon bond between the five-membered and six-membered rings in the core structure produces the fragment at *m*/*z* 253.08577 ([M+H−C_4_H_8_O_4_−C_14_H_16_O_5_]^+^). Subsequent glycosidic bond cleavage and loss of a hydroxyl group results in the fragment at *m*/*z* 107.04909 ([M+H−C_4_H_8_O_4_−C_14_H_16_O_5_−C_5_H_6_O_5_]^+^). Alternatively, cleavage of two glycosidic bonds in the secondary fragment at *m*/*z* 517.31171 followed by the loss of a hydroxyl group yields the fragment at *m*/*z* 267.10138 ([M+H−C_4_H_8_O_4_−C_9_H_14_O_8_]^+^). Cleavage of the glycosidic bond in the secondary fragment at *m*/*z* 517.31171 produces the fragment at *m*/*z* 145.06490 ([M+H−C_4_H_8_O_4_−C_20_H_20_O_7_]^+^). Finally, carbon–carbon bond cleavage between the five- and six-membered rings in the core structure results in the formation of the fragment at *m*/*z* 253.08577 ([M+H−C_18_H_24_O_9_]^+^).

These fragmentation pathways illustrate the complex fragmentation behavior of pueroside B, which involves both glycosidic bond cleavage and core structure modifications. The detailed fragmentation mechanisms provide valuable insights into the structural elucidation of these isomers.

### 2.4. In Vitro Inhibition of α-Glucosidase and α-Amylase

To evaluate the inhibitory effects of compounds **1**–**12** on *α*-amylase and *α*-glucosidase in vitro, acarbose was used as a positive control. The experimental results are presented in Table 2. All tested compounds, except for compound **2**, exhibited some degree of activity. The IC_50_ values for *α*-glucosidase inhibition ranged from 23.25 to 77.87 μM (acarbose: IC_50_ = 27.05 μM), and for *α*-amylase inhibition, the IC_50_ values ranged from 30.89 to 83.62 μM (acarbose: IC_50_ = 36.68 μM). Notably, compound **12** demonstrated the strongest inhibitory effects on both *α*-glucosidase (IC_50_ = 23.25 μM) and *α*-amylase (IC_50_ = 27.05 μM), surpassing acarbose. Compound **11** showed inhibitory effects close to acarbose for *α*-glucosidase (IC_50_ = 32.18 μM) and *α*-amylase (IC_50_ = 38.87 μM). The strong inhibitory activities observed for compounds **11** and **12** can be attributed to their specific structural features, notably the presence of multiple hydroxyl and carbonyl groups, as well as a conjugated double bond system in compound **12**. These functional groups are known to form hydrogen bonds and possibly engage in non-covalent hydrophobic interactions with the active site of enzymes, enhancing binding affinity and stabilizing the enzyme-inhibitor complex. Literature comparisons confirm that such functional groups are crucial for effective *α*-glucosidase and *α*-amylase inhibition, supporting their key role in enzyme binding and inhibition mechanisms [33,34].

Among the three puerosides (compounds **1**–**3**), only compounds **1** and **3** exhibited significant inhibitory activity. Although compounds **1** and **2** are isomers, compound **2** did not show any activity, which may be related to the stereochemistry at the 4-position; the *R* configuration at position 4 appears to confer stronger inhibition of *α*-glucosidase and *α*-amylase. The stereochemical structure of isomers may significantly impact enzyme activity, particularly enzyme inhibition, as observed in this study. Similar findings were reported by He et al., who isolated several novel alkaloids from the endophytic fungus *Aspergillus flavus* GZWMJZ-288. Among these, isomers **1** and **4** demonstrated significant differences in their *α*-glucosidase inhibitory activity, with IC_50_ values of 41.97 and 232.57 µM, respectively [35]. This indicates that stereoisomerism influences the interaction between the enzyme and its ligands, thereby affecting the enzyme’s inhibitory activity. Additionally, García-Chacón et al. found that isomers **1** and **2**, isolated from *Myrciaria dubia*, exhibited particularly potent *α*-glucosidase inhibitory effects, with IC_50_ values of 11.69 µg/mL and 102.69 µg/mL, respectively. The authors noted that the aliphatic moiety of the isomers played a crucial role in their binding mode with the enzyme, impacting the stability and efficacy of the enzyme-ligand complex [36]. These studies underscore the importance of considering isomeric structures in the development of enzyme inhibitors. The six alkaloid compounds (compounds **4**–**9**) also exhibited varying degrees of inhibitory activity, but none were as potent as the positive control. These findings suggest that non-isoflavone compounds extracted from the roots of *P. lobata* may serve as valuable leads for discovering inhibitors of *α*-amylase and *α*-glucosidase. Based on their potent inhibitory effects on *α*-glucosidase and *α*-amylase, these compounds hold potential therapeutic applications in the treatment of diabetes mellitus. *α*-Glucosidase and *α*-amylase are key enzymes involved in carbohydrate digestion, and their inhibition can delay glucose absorption in the intestines, thereby aiding in the control of postprandial blood glucose levels [37]. These compounds may exert their effects by binding to the active sites of *α*-glucosidase and *α*-amylase, competitively blocking the entry of substrates (such as disaccharides and oligosaccharides), and thus slowing their breakdown into glucose. This results in a more gradual and stable increase in postprandial blood glucose levels. Additionally, these compounds may work through complementary mechanisms to further reduce postprandial blood glucose, improve insulin sensitivity, and potentially prevent diabetes-related complications, highlighting their antidiabetic potential.

### 2.5. Molecular Docking Results

**Figure 5:**
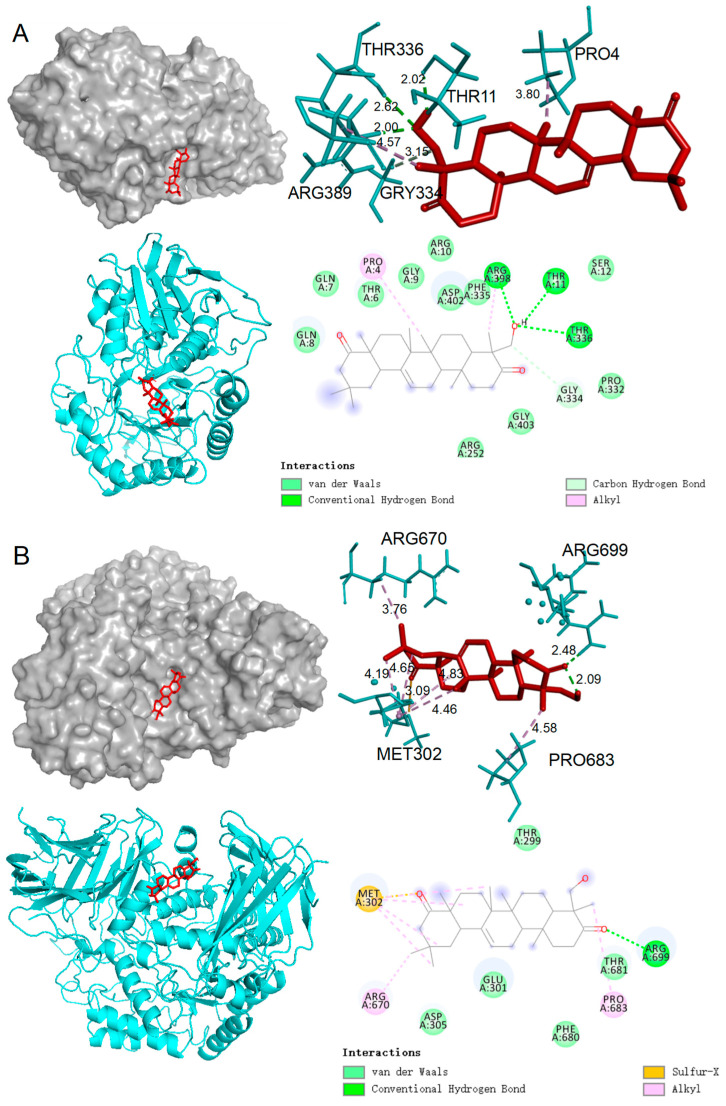
The molecular docking results of compound **12** with enzymes. (**A**) Compound **12** with *α*-glucosidase. (**B**) Compound **12** with *α*-amylase. The left side of each panel shows 3D macro views (upper and lower), the upper right corner presents a 3D local interaction visualization (with the small molecule compound ligand in red and the enzyme protein in blue), and the lower right corner shows a 2D interaction visualization. The PDB ID for *α*-glucosidase is 3W37, and for *α*-amylase, it is 2QV4.

Given the promising in vitro inhibitory effects of compound **12** on *α*-amylase and *α*-glucosidase, molecular docking studies were conducted to investigate their interactions with these enzymes. Molecular docking simulations help elucidate the optimal binding modes between protein receptors and active small molecules. Typically, binding energies below −5 kcal/mol are considered indicative of relatively stable binding [38]. The docking simulations (Table 3, Figure 5) revealed that compound **12** possesses a high binding affinity for both α-amylase and α-glucosidase, with docking scores of −8.15 and −8.81 kcal/mol, respectively (Table 2). These values, significantly lower than the −5 kcal/mol threshold, suggest robust and stable binding interactions between compound **12** and the enzymes, which may underlie its potent inhibitory effects.

Detailed analysis of the docking results shows that compound **12** interacts with the 3W37 protein through multiple key interactions. Specifically, it forms a conventional hydrogen bond with ARG699, which likely plays a critical role in anchoring the compound within the enzyme’s active site. Additionally, van der Waals interactions are observed with several residues, including THR299, THR681, PHE680, GLU301, and ASP305, contributing to the stabilization of the enzyme–ligand complex. Furthermore, alkyl bonds are formed with ARG670 and PRO683, which may enhance the hydrophobic interactions within the binding pocket, further securing the compound in place (Figure 5A). For the 2QV4 protein, compound **12** demonstrates a similarly complex interaction profile. It forms conventional hydrogen bonds with residues THR11, ARG398, and THR336, suggesting a strong and stable attachment to the enzyme. Additionally, alkyl bonds with PRO4 and ARG398, along with a series of van der Waals interactions with residues GLN8, GLN7, THR6, GLY9, ARG10, ASP402, PHE335, SER12, PRO332, GLY403, and ARG252, contribute to the overall stability of the enzyme–ligand complex (Figure 5B). These extensive interactions highlight the ability of compound **12** to effectively occupy the enzyme’s active site, potentially blocking substrate access and inhibiting enzymatic activity.

Collectively, the interactions observed for compound **12** with both 3W37 and 2QV4 proteins primarily involve hydrogen bonds, van der Waals forces, and hydrophobic interactions, which are crucial for the formation of stable enzyme–inhibitor complexes. These molecular interactions are consistent with the significant *α*-glucosidase and *α*-amylase inhibitory activities observed in vitro, supporting the potential of compound **12** as an effective inhibitor for these enzymes.

### 2.6. Comparison of Isolated Compounds and Their Activities with Existing Research on P. lobata

In this study, twelve compounds were successfully isolated and characterized from the roots of *P. lobata*, including three puerosides (compounds **1**–**3**), six alkaloids (compounds **4**–**9**), one phenolic compound (compound **10**), one flavonoid (compound **11**), and one triterpenoid (compound **12**), with Compound **1** (4*R*-pueroside B) being a novel discovery. Compared to previous studies, our research further expands the chemical profile and biological activities of *P. lobata*. Wang et al. focused on flavonoids from *P. lobata*, isolating 53 compounds and demonstrating their potential to lower blood glucose levels through the inhibition of SGLT2, PTP1B, and *α*-glucosidase. While our study also investigates *α*-glucosidase inhibition, it emphasizes puerosides and alkaloids rather than the predominantly studied flavonoids [19]. Similarly, Xiang et al. concentrated on isoflavones from *P. lobata* roots, isolating ten compounds, including two novel isoflavones, which exhibited significant α-glucosidase inhibitory activity with IC_50_ values below 8 µM [20]. Seong et al. investigated triterpenoids in *P. lobata* for their PTP1B inhibitory effects, identifying effective inhibitors such as lupeol and lupenone. Although we did not directly assess PTP1B inhibition, molecular docking studies suggest that the triterpenoid Compound **12** may inhibit α-glucosidase and *α*-amylase through interactions involving hydrogen bonds, van der Waals forces, and hydrophobic interactions with the enzymes’ active sites, consistent with the non-competitive inhibition mode observed by Seong et al. [18]. This study not only extends the chemical profile of *P. lobata* but also supports its potential as a source of natural antidiabetic agents, particularly highlighting the significant inhibitory effects of Compound **12**. Future research should further explore the in vivo antidiabetic effects and mechanisms of these compounds.

## 3. Experimental Section

### 3.1. General Experimental Procedures

ESI-HR-MS was recorded on an Orbitrap Exploris 120 quadrupole electrostatic field orbital well high-resolution mass spectrometer for UPLC-Q-Orbitrap HRMS (Waltham, MA, USA) equipped with a Hypersil GOLD C_18_ column (2.1 × 100 mm, 1.9 μm, ThermoFisher, Waltham, MA, USA). Optical rotations were measured on an MCP 500 polarimeter (Anton Paar, Graz, Austria). CD spectra were obtained using a Chirascan circular dichroism spectrometer (Applied PhotoPhysics, Leatherhead, Surrey, UK). UV spectra were measured with a UV-3600 Plus spectrophotometer (Shimadzu, Kyoto, Japan), and IR spectra were obtained using an IR Affinity-1S spectrophotometer (Shimadzu, Kyoto, Japan). Preparative and semipreparative HPLC were performed on a Shimadzu LC-16 system using a reverse-phase C_18_ column (20 × 250 mm, 5 μm, Shimadzu, Kyoto, Japan). NMR experiments were conducted on a Bruker AVANCE-600 instrument with tetramethylsilane (TMS) as an internal standard, operating at 600 MHz for ^1^H NMR and 150 MHz for ^13^C NMR (Ettlingen, Germany). Enzyme inhibition activity was measured using an Agilent Synergy H1 microplate reader (Agilent Technologies, Santa Clara, CA, USA). Macroporous resin AB-8 was obtained from the Chemical Plant of Nankai University (Tianjin, China). The MCI filler (GEL CHP20P) used for separation was produced by Mitsubishi Chemical (Yokohama, Japan). *α*-Glucosidase (from yeast, EC 3.2.1.20), *α*-amylase (from porcine pancreas), acarbose (purity > 98%), *p*-nitrophenyl-*α*-D-glucopyranoside (*p*-NPG, purity > 98%), 3,5-dinitrosalicylic acid (DNS, purity > 98%), and phosphate-buffered saline (PBS, 0.1 M, pH 6.86) were all purchased from Shanghai Yuanye Bio-Technology Co., Ltd. (Shanghai, China). Analytical-grade reagents were procured from Da Mao Chemical Reagent Co., Ltd. (Tianjin, China), chromatographic-grade reagents from Honeywell Trade (Shanghai) Co., Ltd. (Shanghai, China), and distilled water from Watsons (Hong Kong, China).

### 3.2. Plant Material

The plant material of *P. lobata* (Willd.) Ohwi was collected in July 2023 from Enshi, Hubei Province, China, and identified by Dr. Xinger Ye from Guangdong Pharmaceutical University. A voucher specimen (No. 230112) has been deposited in the Teaching and Experimental Center of Guangdong Pharmaceutical University (Yunfu, China).

### 3.3. Extraction and Isolation

Dried roots of *P. lobata* (5 kg) were pulverized and extracted with 80% ethanol solution three times, each for 7 days. The extracts were concentrated under reduced pressure at 40 °C using a rotary evaporator to yield a total crude extract of 780 g. The crude extract was dissolved in methanol and subjected to column chromatography using an AB-8 macroporous resin. Gradient elution was performed with methanol-water mixtures (20:80, 40:60, 60:40, 80:20, 100:0, *v*/*v*) to obtain five main fractions (Fr.A to Fr.E).

Fraction A (Fr.A) was further separated on an MCI gel column using methanol-water (30:70 to 100:0, *v*/*v*) as the eluent, yielding six subfractions (Fr.A-1 to Fr.A-6). Subfraction Fr.A-1 was purified by preparative HPLC using acetonitrile-water (40:60, *v*/*v*) isocratic elution to obtain compounds **1** (3.2 mg) and 2 (5.6 mg). Subfraction Fr.A-2 was purified by isocratic elution with methanol-water (40:60, *v*/*v*) to obtain compound **3** (4.6 mg).

Fraction B (Fr.B) was separated on an MCI gel column using methanol-water (40:60 to 100:0, *v*/*v*) as the eluent, and TLC analysis yielded five subfractions (Fr.B-1 to Fr.B-5). Subfraction Fr.B-1 was purified by isocratic elution with methanol-water (35:65, *v*/*v*) to obtain compound **7** (3.7 mg). Subfraction Fr.B-2 was purified by isocratic elution with methanol-water (40:60, *v*/*v*) to obtain compound **8** (12.6 mg). Subfraction Fr.B-3 was first eluted with methanol-water (40:60, *v*/*v*) and then with acetonitrile-water (40:60, *v*/*v*) using preparative HPLC to isolate compound **6** (5.6 mg). Fraction Fr.B-4 was subjected to two preparative HPLC runs with acetonitrile-water (40:60, *v*/*v*) to yield compounds **9** (4.6 mg) and 5 (12.5 mg). Fraction Fr.B-5 was first eluted with acetonitrile-water (40:60, *v*/*v*) and then with methanol-water (35:65, *v*/*v*) using preparative HPLC to obtain compound **4** (7.1 mg).

Fraction D (Fr.D) was eluted with methanol-water (40:60, *v*/*v*) using preparative HPLC to yield subfractions Fr.D-1 to Fr.D-4. Subfraction Fr.D-1 was eluted with acetonitrile-water (55:45, *v*/*v*) to obtain compound **10** (8.1 mg). Subfraction Fr.D-2 was eluted with acetonitrile-water (60:40, *v*/*v*) using preparative HPLC, followed by a second elution with methanol-water (35:65, *v*/*v*) to obtain compound **12** (7.2 mg). Subfraction Fr.D-4 was eluted with acetonitrile-water (60:40, *v*/*v*) using preparative HPLC to obtain compound **11** (6.4 mg).

### 3.4. Structure Identification of Compounds

The structure of the novel compound was elucidated using ESI-HR-MS, 1D and 2D NMR, and CD spectroscopy. The structures of the known compounds were identified by NMR or ESI-HR-MS and compared with the relevant literature data.

### 3.5. LC-MS Analysis of Compounds ***1*** and ***2***

The LC-MS analysis of compounds **1** and **2** was performed under the following conditions. The mobile phase consisted of 0.1% formic acid in acetonitrile (A) and 0.1% formic acid in water (B). Gradient elution was carried out with the following program: 95–85% B from 0 to 5 min, 80–65% B from 5 to 30 min, 65–18% B from 30 to 40 min, 18–12% B from 40 to 47 min, and 12–5% B from 47 to 50 min. The flow rate was maintained at 0.3 mL/min, with an injection volume of 2 μL and a column temperature of 35 °C. The detection wavelength for the diode array detector (DAD) was set at 330 nm. Mass spectrometry was conducted with positive and negative ion switching using full scan/dd-MS2 mode. The ion source was HESI (Heated Electrospray Ionization) with a spray voltage of +3.5 kV for positive ions and −2.8 kV for negative ions. The capillary temperature was set at 325 °C, and the auxiliary gas heater temperature was 350 °C. The mass range for scanning was *m*/*z* 100–1500, with sheath gas, auxiliary gas, and sweep gas (all nitrogen) flow rates at 50 Arb, 8 Arb, and 1 Arb, respectively. The resolution was set to 60,000 for full scans and 15,000 for dd-MS2 scans, with normalized collision energy (NCE) levels at 20%, 40%, and 60%.

### 3.6. Spectral and Physical Data of Compounds

Compound **1**: 4*R*-pueroside B. White amorphous powder; ${\left[\alpha \right]}_{D}^{20}$ = −0.0041 (c 0.01, MeOH); IR (KBr): 3381, 2882, 1721, 1609, 1508, 1431, 1231, 1074, 1039 cm^−1^; CD (c 0.01, MeOH): Δε 228 −0.75, Δε 252 +0.45, Δε 286 −0.97; ^1^H NMR (DMSO-*d*_6_, 600 MHz) and ^13^C NMR (DMSO-*d*_6_, 150 MHz) data (see Table 1); ESI-HR-MS (*m*/*z*): 637.21307 ([M + H]^+^, C_30_H_37_O_15_^+^; calc. 637.21270). The relevant spectra for these data can be found in Supplementary Materials Figures S1–S10.

Compound **2**: 4*S*-pueroside B. White amorphous powder; CD (c 0.01, MeOH): Δε 229 +0.66, Δε 246 −3.22, Δε 286 +3.07; ESI-HR-MS (*m*/*z*): 637.21277 ([M + H]^+^, C_30_H_37_O_15_^+^; calc. 637.21270); ^1^H NMR (400 MHz, DMSO-*d*_6_) *δ*_H_ 2.60–2.66 (2H, m, H-4a), 3.84 (3H, s, OCH_3_), 6.35 (1H, br s, H-2), 6.05 (1H, m, H-4), 7.07 (2H, d, *J* = 8.0 Hz, H-2′, H-6′), 6.90 (2H, d, *J* = 4.0 Hz, H-3′,H-5′), 6.91 (1H, br s, H-3″), 6.73 (1H, br d, *J* = 8.0 Hz, H-5″), 7.58 (l H, d, *J* = 4.0 Hz, H-6″), 5.05 (2H, m, glc-H), 3.16–3.74 (12H, m, glc-H). The relevant spectra for these data can be found in Supplementary Materials Figures S13–S15.

Compound **3**: (−)-puerol B-2-O-glucopyranoside. White amorphous powder; ESI-HR-MS (*m*/*z*): 475.15961 ([M + H]^+^, C_24_H_27_O_10_^+^; calc. 475.15987); ^1^H NMR (400 MHz, DMSO-*d*_6_) *δ*_H_ 6.34 (1H, d, *J* = 1.0 Hz, H-2), 6.02 (1H, m, H-4), 6.95 (2H, d, *J* = 8.0 Hz, H-2′, H-6′), 6.62 (2H, d, *J* = 8.0 Hz, H-3′, H-5′), 6.88 (1H, d, *J* = 2.0 Hz, H-3″), 6.72(1H, dd, *J* = 2.0, 8.0 Hz, H-5″), 7.57 (1H, d, *J* = 8.0 Hz, H-6″), 5.06 (1H, d, *J* = 7.0 Hz, glc-H-1), 3.83 (3H, s, OCH_3_), 3.03 (1H, dd, *J* = 3.0, 14.0 Hz, H-4a), 2.50 (1H, *J* = 7.0, 14.0 Hz, H-4a), 3.03–3.76 (6H, m, glc-H). The relevant spectra for these data can be found in Supplementary Materials Figures S16 and S17.

Compound **4**: dauricine. White amorphous powder; ^1^H NMR (400 MHz, CDCl_3_) *δ*_H_ 7.97 (2H, d, *J* = 8.4 Hz, H-11′,13′), 6.82 (1H, d, *J* = 8.0 Hz, H-13), 6.81 (2H, d, *J* = 8.4 Hz, H-10′,14′), 6.78 (1H, dd, *J* = 2.0,8.0 Hz, H-14), 6.53 (1H, d, *J* = 2.0 Hz, H-10), 6.53 (1H, s, H-5), 6.48 (1H, s, H-5′), 6.00 (1H, s, H-8), 5.97 (1H, s, H-8′), 3.80 (3H, s, 6-OCH_3_), 3.78 (3H, s, 6′-OCH_3_), 3.68 (1H, m, H-1), 3.64 (1H, m, H-1′), 3.55 (3H, s, 7-OCH_3_), 3.53 (3H, s, 7′-OCH_3_), 3.18, 2.68 (each 1H, m, H-4), 3.13 (2H, m, H-3), 3.05, 2.75 (each 1H, m, H-4′), 2.85 (2H, m, H-9), 2.78, 2.70 (each 1H, m, H-3′), 2.56 (2H, m, H-9′), 2.48 (3H, s, N-CH_3_), 2.45 (3H, s, N′-CH_3_); ^13^C NMR (100 MHz, CDCl_3_) *δ*_C_ 64.6 (C-1), 64.5 (C-1′), 46.4 (C-3), 46.4 (C-3′), 25.1 (C-4), 24.9 (C-4′), 125.7 C-4a), 125.6 (C-4a′), 110.9 (C-5), 110.9 (C-5′), 147.2 (C-6), 147.1 (C-6′), 146.4 (C-7), 146.2 (C-7′), 111.0 (C-8, C-8′), 125.8 (C-8a, C-8a′), 134.4 (C-9), 131.7 (C-9′), 40.5 (C-9a), 40.1 (C-9a′), 116.4 (C-10), 128.7 (C-10′), 143.3 (C-11), 111.1 (C-11′), 146.1 (C-12), 155.5 (C-12′), 120.9 (C-13), 117.2 (C-13′), 130.8 (C-14,C-14′), 55.6 (6-OCH_3_), 55.6 (6′-OCH_3_), 55.4 (7-OCH_3_), 55.4 (7′-OCH_3_), 42.3 (N-CH_3_), 42.3 (N′-CH_3_). The relevant spectra for these data can be found in Supplementary Materials Figures S18 and S19.

Compound **5**: daurisoline. White amorphous powder; ^1^H NMR (400 MHz, DMSO-*d*_6_) *δ*_H_ 7.04 (2H, d, *J* = 8.4 Hz, H-10′, 14′), 6.66 (1H, s, H-13), 6.65 (1H, s, H-14), 6.78 (2H, d *J* = 8.4 Hz, H-11′,13′), 6.64 (1H, s, H-5), 6.61 (1H, s, H-10), 6.52 (1H, s, H-5′), 6.37 (1H, s, H-8), 6.34 (1H, s, H-8′), 3.69 (3H, s, 7′-OCH_3_), 3.68 (3H, s, 6′-OCH_3_), 3.64 (1H, m, H-1′), 3.63 (3H, s, 6-OCH_3_), 3.61 (1H, m, H-1), 2.49 (3H, s, N2′-CH_3_), 2.48 (3H, s, N2-CH_3_); ^13^C NMR (100 MHz, DMSO-*d*_6_) *δ*_C_ 64.4 (C-1), 64.5 (C-1′), 49.1 (C-3), 49.1 (C-3′), 25.1 (C-4), 25.6 (C-4′), 126.4 (C-4a), 126.6 (C-4a′), 114.9 (C-5), 112.1 (C-5′), 146.2 (C-6), 147.5 (C-6′), 142.2 (C-7), 147.4 (C-7′), 115.9 (C-8), 111.7 (C-8′), 130.0 (C-8a), 129.8 (C-8a′), 42.8 (C-9), 43.0 (C-9′), 131.6 (C-9a), 133.7 (C-9a′), 123.4 (C-10), 130.9 (C-10′), 144.5 (C-11), 116.9 (C-11′), 146.8 (C-12), 156.6 (C-12′), 115.9 (C-13), 116.9 (C-13′), 125.2 (C-14), 130.9 (C-14′), 55.8 (6-OCH_3_), 55.8 (6′-OCH_3_), 55.9 (7′-OCH_3_), 46.7 (N2-CH_3_), 47.7 (N2′-CH_3_). The relevant spectra for these data can be found in Supplementary Materials Figures S20 and S21.

Compound **6**: chelerythrine. Orange amorphous powder; ^1^H NMR (400 MHz, MeOD) *δ*_H_ 9.96 (1H, s, H-6), 8.63 (1H, d, *J* = 8.8 Hz, H-10), 8.60 (1H, d, *J* = 9.2 Hz, H-11), 8.18 (1H, d, *J* = 8.8 Hz, H-9), 8.17 (1H, d, *J* = 9.2 Hz, H-12), 8.16 (1H, s, H-4), 7.52 (1H, s, H-1), 6.28 (2H, s, –OCH_2_O-2, 3), 4.29 (3H, s, 7-OCH_3_), 4.14 (3H, s, 8-OCH_3_), 4.98 (3H, s, N-CH_3_); ^13^C NMR (100 MHz, MeOD) *δ*_C_ 107.0 (C-1), 150.9 (C-2), 150.7 (C-3), 105.1 (C-4), 121.7 (C-4a), 132.6 (C-4b), 152.0 (C-6), 119.9 (C-6a), 147.4 (C-7), 151.7 (C-8), 127.3 (C-9), 120.8 (C-10), 129.9 (C-10a), 127.0 (C-10b), 119.4 (C-11), 133.3 (C-12), 134.2 (C-12a), 104.3 (–OCH_2_O-2, 3), 62.8 (7-OCH_3_), 57.5 (8-OCH_3_), 52.9 (N-CH_3_). The relevant spectra for these data can be found in Supplementary Materials Figures S22 and S23.

Compound **7**: stepholidine. Yellow amorphous powder; ^1^H NMR (400 MHz, DMSO-*d*_6_) *δ*_H_ 6.72 (1H, s, H-1), 6.70 (1H, d, *J* = 8.2 Hz, H-11), 6.69 (1H, d, *J* = 8.2 Hz, H-12), 6.63 (1H, s, H-4), 4.04 (1H, d, *J* = 16.2 Hz, Ha-8), 3.74 (3H, s, 3-OCH_3_), 3.72 (3H, s, 9-OCH_3_), 3.15 (1H, m, Hb-8), 2.45–3.14 (4H, m, H-5, 6), 2.86 (1H, m, H-13a), 2.56 (2H, m, H-13); ^13^C NMR (100 MHz, DMSO-*d*_6_) *δ*_C_ 112.4 (C-1), 147.3 (C-2), 144.6 (C-3), 112.4 (C-4), 125.8 (C-4b), 130.0 (C-4a), 28.5 (C-5), 51.2 (C-6), 53.6 (C-8), 128.3 (C-8a), 143.3 (C-9), 146.1 (C-10), 112.4 (C-11), 123.7 (C-12), 125.8 (C-12a), 35.8 (C-13), 59.2 (C-13a), 58.7 (3-OCH_3_), 55.5 (9-OCH_3_). The relevant spectra for these data can be found in Supplementary Materials Figures S24 and S25.

Compound **8**: acutumine. White amorphous powder; ^1^H NMR (400 MHz, DMSO-*d*_6_) *δ*_H_ 6.18 (1H, 1-OH), 5.43 (1H, s, H-3), 4.58 (1H, s, H-1), 4.48 (1H, dd, *J* = 12.0, 7.0 Hz, H-10), 3.98 (3H, s, 8-OCH_3_), 3.86 (3H, s, 7-OCH_3_), 3.55 (3H, s, 2-OCH_3_), 2.63 (1H, m, H-15), 2.48–2.36 (2H, m, H-14, H-15), 2.28 (1H, d, *J* = 7.0 Hz, H-9), 2.26 (3H, s, N-CH_3_), 2.23 (1H, m, H-5), 2.11 (1H, m, H-5), 1.49 (1H, m, H-14); ^13^C NMR (100 MHz, DMSO-*d*_6_) *δ*_C_ 69.4 (C-1), 188.5 (C-2), 104.9 (C-3), 200.6 (C-4), 45.7 (C-5), 192.1 (C-6), 137.8 (C-7), 159.4 (C-8), 40.4 (C-9), 56.9 (C-10), 67.1 (C-11), 52.4 (C-12), 71.9 (C-13), 37.5 (C-14), 51.0 (C-15), 59.0 (2-OCH_3_), 59.8 (7-OCH_3_), 60.2 (8-OCH_3_), 36.0 (N-CH_3_). The relevant spectra for these data can be found in Supplementary Materials Figures S26 and S27.

Compound **9**: sinomenine. White amorphous powder; ^1^H NMR (400 MHz, CDCl_3_) *δ*_H_ 6.61 (1H, d, *J* = 8.0 Hz, H-2), 6.51 (1H, d, *J* = 8.0 Hz, H-1), 5.45 (1H, d, *J* = 1.8 Hz, H-8), 4.33 (1H, d, *J* = 15.2 Hz, H-5e), 3.78 (3H, s, 3-OCH_3_), 3.47 (3H, s, 7-OCH_3_), 3.17 (1H, t, *J* = 4.2 Hz, H-9), 2.98 (2H, m, H-14, H-10e), 2.69 (1H, m, H-10a), 2.52 (1H, m, H-5a), 2.49 (1H, m, H-16), 2.43 (3H, s, N-CH_3_), 2.05 (1H, m, H-16), 1.87 (2H, m, H-15); ^13^C NMR (100 MHz, CDCl_3_) *δ*_C_ 118.3 (C-1), 109.1 (C-2), 145.1 (C-3), 144.9 (C-4), 49.3 (C-5), 194.1 (C-6), 152.5 (C-7), 115.3 (C-8), 54.9 (C-9), 24.4 (C-10), 130.6 (C-11), 122.8 (C-12), 40.6 (C-13), 46.0 (C-14), 36.1 (C-15), 47.2 (C-16), 56.8 (3-OCH_3_), 56.1 (7-OCH_3_), 42.9 (N-CH_3_). The relevant spectra for these data can be found in Supplementary Materials Figures S28 and S29.

Compound **10**: 1-hydroxy-2-propanone. Yellow solid; ESI-HR-MS (*m*/*z*): 289.10690 ([M + H]^+^, C_16_H_17_O_5_^+^; calc. 289.10705); ^1^H NMR (400 MHz, MeOD) *δ*_H_ 7.03 (1H, d, *J* = 8.2 Hz, H-6”), 6.83 (2H, d, *J* = 8.6 Hz, H-2′, 6′), 6.65 (2H, d, *J* = 8.6 Hz, H-3, 5′), 6.45 (1H, d, *J* = 2.6 Hz, H-3”), 6.40 (1H, dd, *J* = 2.6, 8.2 Hz, H-5”) 5.39 (1H, s, H-1), 4.91 (1H, s, 1-OH), 3.75 (3H, s, OCH_3_), 3.51 (1H, d, *J* = 15.8 Hz, 3-CH). The relevant spectra for these data can be found in Supplementary Materials Figures S30 and S31.

Compound **11**: hydroxytuberosone. Yellow amorphous powder; ESI-HR-MS (*m*/*z*): 355.11716 ([M + H]^+^, C_20_H_19_O_6_^+^; calc. 355.11761); ^1^H NMR (400 MHz, (CD_3_)_2_CO) *δ*_H_ 7.13 (1H, s, H-7), 6.85 (1H, d, *J* = 6.8 Hz, H-1), 6.39 (1H, d, *J* = 6.8 Hz, H-4′), 6.11 (1H, s, H-10), 6.06 (1H, dd, *J* = 7.6, 1.2 Hz, H-2), 5.60 (1H, d, *J* = 7.6 Hz, H-3′), 5.42 (1H, d, *J* = 1.2 Hz, H-4), 5.03 (1H, d, *J* = 6.8 Hz, H-6a), 4.85 (1H, s, H-11a), 4.44 (1H, d, *J* = 6.8 Hz, H-6b), 1.38 (3H, s, –CH_3_), 1.37 (3H, s, –CH_3_). The relevant spectra for these data can be found in Supplementary Materials Figures S32 and S33.

Compound **12**: melilotigenin B. White amorphous powder; ESI-HR-MS (*m*/*z*): 455.35162 ([M + H]^+^, C_30_H_47_O_3_^+^; calc. 455.35197); ^1^H NMR (400 MHz, CDCl_3_) *δ*_H_ 1.92 (1H, m, H-1a), 1.58 (1H, m, H-1b), 2.53 (1H, m, H-2*α*), 2.45 (1H, m, H-2*β*), 0.98 (1H, m, H-5), 1.58 (1H, m, H-6a), 1.26 (1H, m, H-6b), 1.44 (1H, m, H-7a), 1.34 (1H, m, H-7b), 1.68 (1H, m, H-9), 1.94 (2H, m, H-11), 5.32 (1H, m, H-12), 1.76 (1H, m, H-15a), 1.02 (1H, m, H-15b), 1.97 (1H, m, H-16a), 1.24 (1H, m, H-16b), 2.35 (1H, m, H-18), 2.10 (1H, m, H-19a), 1.35 (1H, m, H-19b), 2.45 (1H, m, H-21a), 2.02 (1H, m, H-21b), 1.27 (1H, m, H-23), 3.97 (1H, dd, *J* = 2.0, 8.0 Hz, H-24a), 3.48 (1H, dd, *J* = 6.0, 10.0 Hz, H-24b), 1.00 (9H, s, –CH_3_-25, 26, 27), 1.24 (1H, m, H-28), 0.98 (3H, s, H-29), 0.86 (3H, s, H-30). The relevant spectra for these data can be found in Supplementary Materials Figures S34 and S35.

### 3.7. α-Glucosidase Inhibition Assay

The *α*-glucosidase inhibition assay was performed as described in the literature [39], with acarbose as the positive control. *α*-Glucosidase (5 U/mL) was incubated with test sample solutions (5.00, 10.00, 20.00, 40.00, 60.00, 80.00, and 100.00 μM) at 37 °C for 10 min. Then, 400 μL of *p*-NPG solution (5 mM) was added to initiate the enzymatic reaction, which continued to incubate at 37 °C for 20 min. The reaction was terminated by placing the mixture in a −20 °C freezer for 10 min. Absorbance was measured at 405 nm using a microplate reader. The inhibition rate was calculated based on three parallel measurements, and the IC_50_ value, representing the concentration required to inhibit 50% of the enzyme activity, was determined.

### 3.8. α-Amylase Inhibition Assay

The *α*-amylase inhibition assay was conducted using a colorimetric method described in the literature [39], with acarbose as the positive control. Test sample solutions at various concentrations (5.00, 10.00, 20.00, 40.00, 60.00, 80.00, and 100.00 μM) were preincubated with *α*-amylase (2 U/mL) at 37 °C for 10 min. The reaction was terminated by adding 200 μL of DNS reagent and boiling the mixture at 100 °C for 5 min to inactivate the enzyme. After cooling to room temperature, 1 mL of distilled water was added for dilution, and absorbance was measured at 540 nm. The inhibition rate was calculated based on three parallel measurements, and the IC_50_ value, representing the concentration required to inhibit 50% of the enzyme activity, was determined.

### 3.9. Molecular Docking

Molecular docking simulations were conducted to assess the binding affinity and interaction modes of compound **12** with the active sites of *α*-glucosidase and *α*-amylase. The three-dimensional structures of *α*-glucosidase (PDB ID: 3W37) and *α*-amylase (PDB ID: 2QV4) were obtained from the RCSB Protein Data Bank [40]. The 3D structure of compound **12** was retrieved from PubChem and saved in SDF format. The enzyme structures were processed in PyMOL, where water molecules, ligands, and non-essential atoms were removed. The SDF format of compound **12** was converted to MOL2 format using OpenBabel. The enzyme proteins were preprocessed using AutoDockTools 1.5.7, which involved adding hydrogen atoms, calculating Gasteiger charges, and assigning AD4 atom types [41]. The preprocessing of compound **12** included selecting the molecular root, detecting and expanding the root, and defining rotatable bonds using AutoDockTools 1.5.7. The docking simulations were performed using autogrid4 and autodock4 to calculate the binding energies. Finally, the interactions between compound **12** and the target enzymes were visualized in both 3D and 2D using Discovery Studio 2019 Client and PyMOL.

## 4. Conclusions

In this study, twelve chemical constituents were isolated from the roots of *P. lobata*, including three puerosides (compounds **1**–**3**), six alkaloids (compounds **4**–**9**), and three other types of compounds (compounds **10**–**12**). Among these, compound **1** is a novel compound. The structures of all isolated compounds were determined through various spectroscopic analyses. The absolute configurations of the pueroside isomers (compounds **1** and **2**) were established for the first time using CD spectroscopy, and detailed secondary mass spectrometry fragmentation patterns were analyzed. Enzyme inhibition assays demonstrated that, except for compound **2**, all other compounds exhibited varying degrees of *α*-glucosidase and *α*-amylase inhibitory activity. Structure–activity relationship analysis revealed that the configuration at position 4 in pueroside B significantly influences the inhibitory activity against *α*-glucosidase and *α*-amylase, with the R configuration exhibiting stronger inhibition. Among all isolated compounds, compounds **11** and **12** showed notable inhibitory activities against both *α*-glucosidase and *α*-amylase, with compound **12** outperforming the positive control, acarbose. Molecular docking studies indicated that compound **12** could bind to the active sites of *α*-glucosidase and *α*-amylase through hydrogen bonds, van der Waals forces, and hydrophobic interactions, thereby exerting their inhibitory effects. Consequently, the chemical constituents from *P. lobata* could be potential natural sources of *α*-amylase and *α*-glucosidase inhibitors, with compound **12** being a promising candidate for further investigation.

## Figures and Tables

**Table 1:** The ^1^H NMR (600 MHz) and ^13^C NMR (150 MHz) data for compound **1** in DMSO-*d*_6_.

No.	*δ*_H_ (ppm, *J* in Hz)	*δ*_C_ (ppm), Type
1	-	172.9, C
2	6.47 (br s)	114.8, CH
3	-	163.7, C
4	6.02 (m)	82.1, CH
4a	2.71 (dd, 14.7, 6.5)	38.1, CH_2_
	3.15 (m)	
1′	-	112.0, C
2′	-	157.1, C
3′	6.90 (d, 2.4)	101.4, CH
4′	-	162.6, C
5′	6.74 (dd, 8.7, 2.4)	108.0, CH
6′	7.47 (d, 8.7)	130.8, CH
1″	-	128.7, C
2″, 6″	6.94 (d, 8.6)	130.6, CH
3″, 5″	6.87 (d, 8.6)	115.5, CH
4″	-	156.2, C
Glc-1‴	4.81 (d, 7.6)	100.3, CH
2‴	3.32 (m)	73.2, CH
3‴	3.44 (m)	77.4, CH
4‴	3.15 (m)	69.9, CH
5‴	3.24 (m)	76.9, CH
6‴	3.67 (m)	60.8, CH_2_
	3.45 (m)	
Glc-1⁗	5.12 (d, 7.7)	99.9, CH
2⁗	3.20 (m)	73.2, CH
3⁗	3.14 (m)	76.9, CH
4⁗	3.32 (m)	69.6, CH
5⁗	3.33 (m)	76.6, CH
6⁗	3.73 (m)	60.6, CH_2_
	3.45 (m)	
4′-OCH_3_	3.84 (s)	55.5, CH_3_

**Table 2:** The in vitro *α*-glucosidase and *α*-amylase inhibitory activities of compounds **1**−**12**.

Compounds	IC_50_ (μM) ^1^	
	*α*-Glucosidase	*α*-Amylase
**1**	61.33 ± 1.15	81.65 ± 1.72
**2**	>100	>100
**3**	72.87 ± 0.79	83.62 ± 1.15
**4**	44.15 ± 1.03	60.88 ± 1.68
**5**	45.23 ± 1.27	66.39 ± 2.31
**6**	52.30 ± 1.59	71.41 ± 1.24
**7**	58.77 ± 1.55	78.22 ± 1.98
**8**	66.54 ± 2.03	80.69 ± 1.85
**9**	57.62 ± 0.88	66.75 ± 1.45
**10**	43.58 ± 0.76	63.57 ± 1.37
**11**	32.18 ± 0.71	38.87 ± 1.08
**12**	23.25 ± 0.38	30.89 ± 0.57
Acarbose ^2^	27.05 ± 0.56	36.68 ± 0.77

^1^ IC_50_ values are presented as means ± standard deviation (SD, n = 3); ^2^ Acarbose is used as the positive control.

**Table 3:** Molecular docking binding energies of compounds **11** and **12** with *α*-glucosidase and *α*-amylase.

Compounds	PDB ID ^1^	Binding Energy (kcal/mol)	Compounds	PDB ID ^1^	Binding Energy (kcal/mol)
**1**	3W37	−5.71	**7**	3W37	−6.95
	2QV4	−5.43		2QV4	−6.55
**2**	3W37	−2.00	**8**	3W37	−5.50
	2QV4	−3.80		2QV4	−5.36
**3**	3W37	−5.95	**9**	3W37	−6.20
	2QV4	−5.62		2QV4	−5.87
**4**	3W37	−7.26	**10**	3W37	−6.89
	2QV4	−7.35		2QV4	−5.28
**5**	3W37	−6.59	**11**	3W37	−7.04
	2QV4	−6.16		2QV4	−6.88
**6**	3W37	−7.65	**12**	3W37	−8.15
	2QV4	−6.03		2QV4	−8.81

^1^ The PDB ID of α-glucosidase was 3W37, and that of α-amylase was 2QV4.

## Data Availability

Data are contained within the article and Supplementary Materials.

## Supplementary Materials

The following supporting information can be downloaded at: https://www.mdpi.com/article/10.3390/ijms25179602/s1.
