# Supplementary material for: Isolation and Characterization of Novel Pueroside B Isomers and Other Bioactive Compounds from Pueraria lobata Roots: Structure Elucidation, α-Glucosidase, and α-Amylase Inhibition Studies
Source: Int J Mol Sci. 2024 Sep 4;25(17):9602. doi: 10.3390/ijms25179602 (PMC11395397; doi:10.3390/ijms25179602)
Supplement: Supplementary file 1 [file ijms-25-09602-s001.zip › ijms-3155629-supplementary.pdf]

Isolation and Characterization of Novel Pueroside B Isomers and Other Bioactive Compounds from *Pueraria lobata* Roots: Structure Elucidation, *α*-Glucosidase, and *α*-Amylase Inhibition Studies

Wei Dai [1,2], Manqiu Lei [3], Qiuxiong Yin [4], Haijun Nan [3,*], and Guoqiang Qian [4,*]

[1] Teaching and Experimental Center, Guangdong Pharmaceutical University, Guangzhou 510006, China
[2] Comprehensive Experimental Teaching Center of Traditional Chinese Medicin, Yunfu Campus, Guangdong Pharmaceutical University, Yunfu 527500, China
[3] School of Chinese Materia Medica, Guangdong Pharmaceutical University, Guangzhou 510006, China
[4] School of Chinese Medicine, Guangdong Pharmaceutical University, Guangzhou 510006, China
* Correspondence: nanhj@gdpu.edu.cn (H.N.); tgqqian@gdpu.edu.cn (G.Q.)

Contents

Figure S1 ESI-HR-MS Spectrum of **1**
Figure S2 IR Spectrum of **1**
Figure S3 UV Spectrum of **1**
Figure S4 CD Spectrum of **1**
Figure S5 ^{1}H-NMR Spectrum of **1**
Figure S6 ^{13}C-NMR Spectrum of **1**
Figure S7 HSQC Spectrum of **1**
Figure S8 HMBC Spectrum of **1**
Figure S9 ^{1}H-^{1}H COSY Spectrum of **1**
Figure S10 NOESY Spectrum of **1**
Figure S11 HPLC chromatogram of **1** (4*R*-pueroside) and **2** (4*S*-pueroside)
Figure S12 MS / MS fragment diagram of **1**
Figure S13 CD Spectrum of **2**
Figure S14 ESI-HR-MS Spectrum of **2**
Figure S15 ^{1}H-NMR Spectrum of **2**
Figure S16 ESI-HR-MS Spectrum of **3**
Figure S17 ^{1}H-NMR Spectrum of **3**
Figure S18 ^{1}H-NMR Spectrum of **4**
Figure S19 ^{13}C-NMR Spectrum of **4**
Figure S20 ^{1}H-NMR Spectrum of **5**
Figure S21 ^{13}C-NMR Spectrum of **5**
Figure S22 ^{1}H-NMR Spectrum of **6**
Figure S23 ^{13}C-NMR Spectrum of **6**
Figure S24 ^{1}H-NMR Spectrum of **7**
Figure S25 ^{13}C-NMR Spectrum of **7**
Figure S26 ^{1}H-NMR Spectrum of **8**
Figure S27 ^{13}C-NMR Spectrum of **8**
Figure S28 ^{1}H-NMR Spectrum of **9**
Figure S29 ^{13}C-NMR Spectrum of **9**
Figure S30 ESI-HR-MS Spectrum of **10**
Figure S31 ^{1}H-NMR Spectrum of **10**
Figure S32 ESI-HR-MS Spectrum of **11**
Figure S33 ^{1}H-NMR Spectrum of **11**
Figure S34 ESI-HR-MS Spectrum of **12**
Figure S35 ^{1}H-NMR Spectrum of **12**

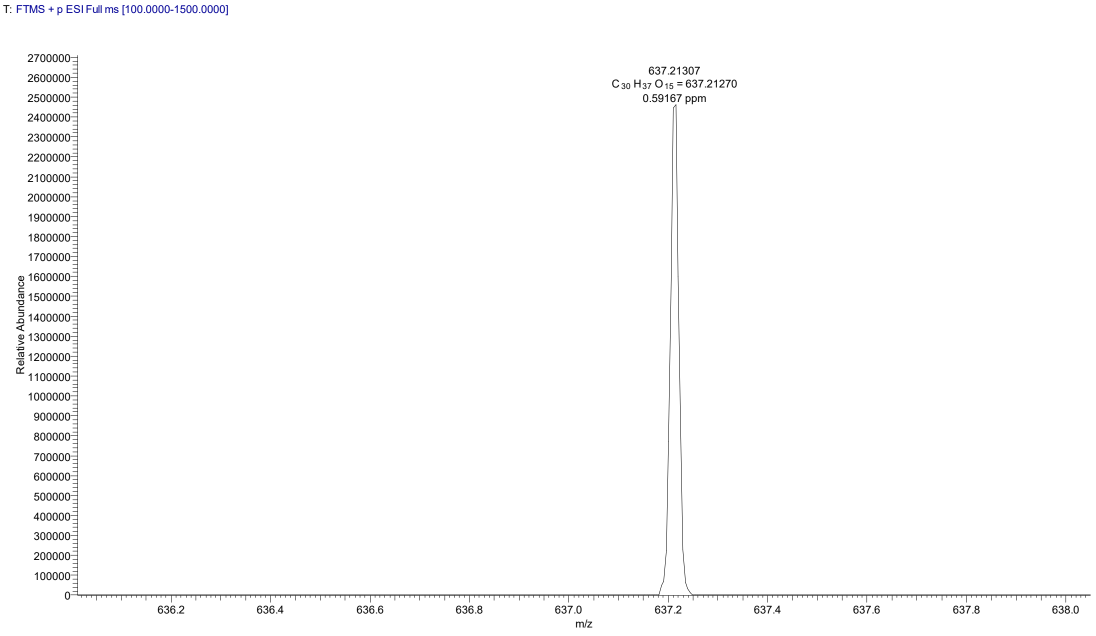

Figure S1 ESI-HR-MS Spectrum of **1**

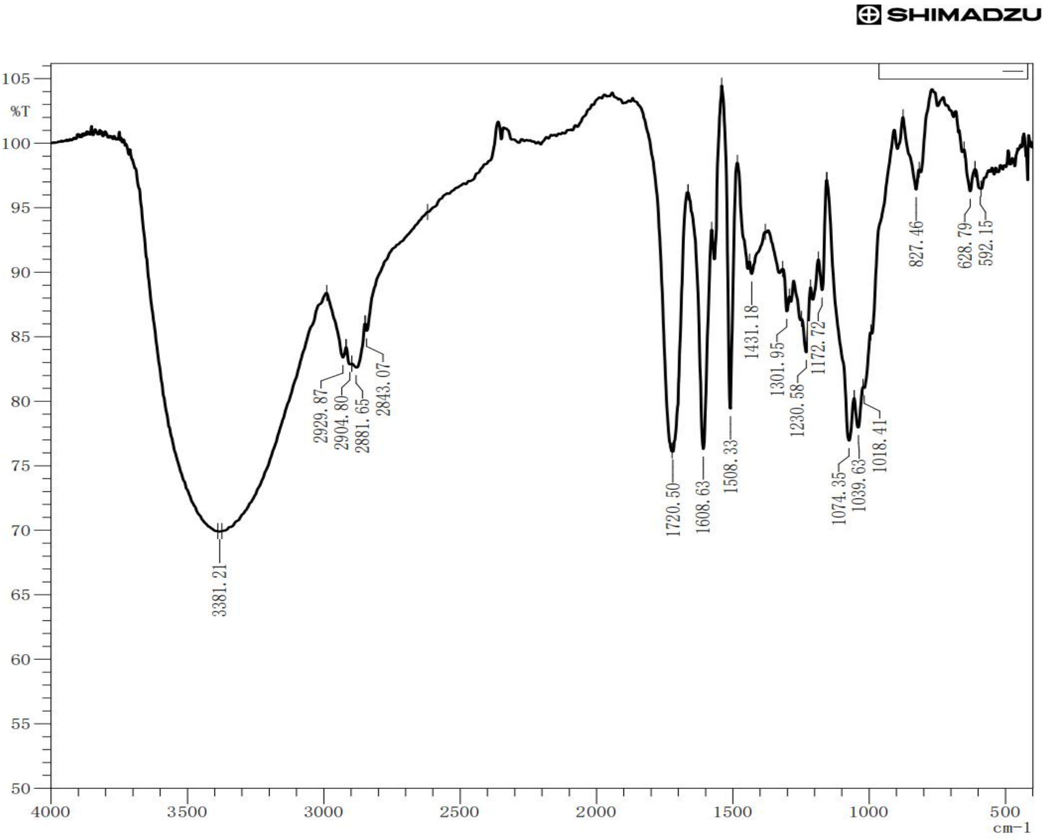

Figure S2 IR Spectrum of **1**

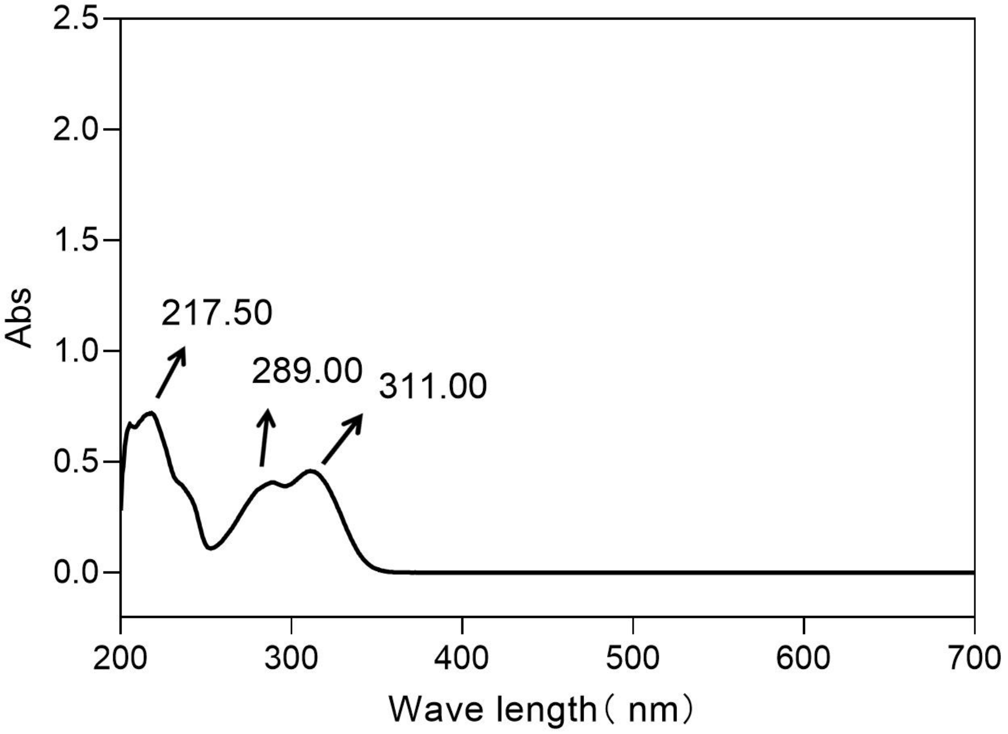

Figure S3 UV Spectrum of **1**

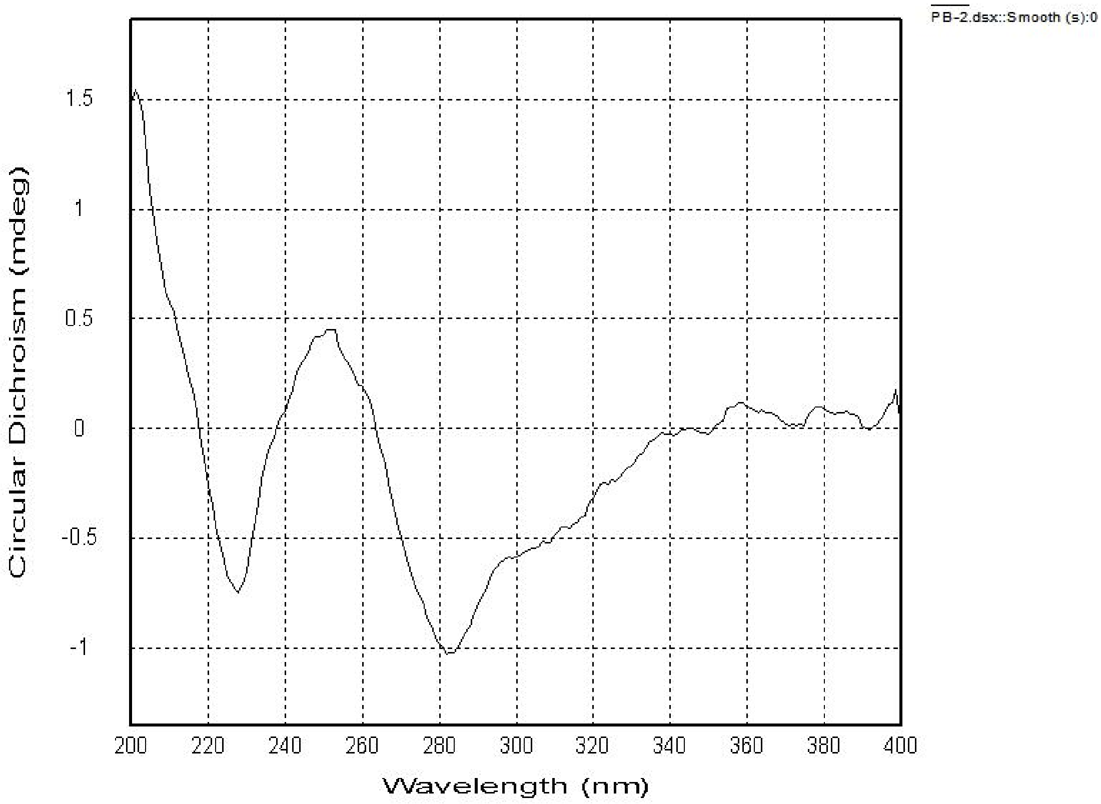

Figure S4 CD Spectrum of **1**

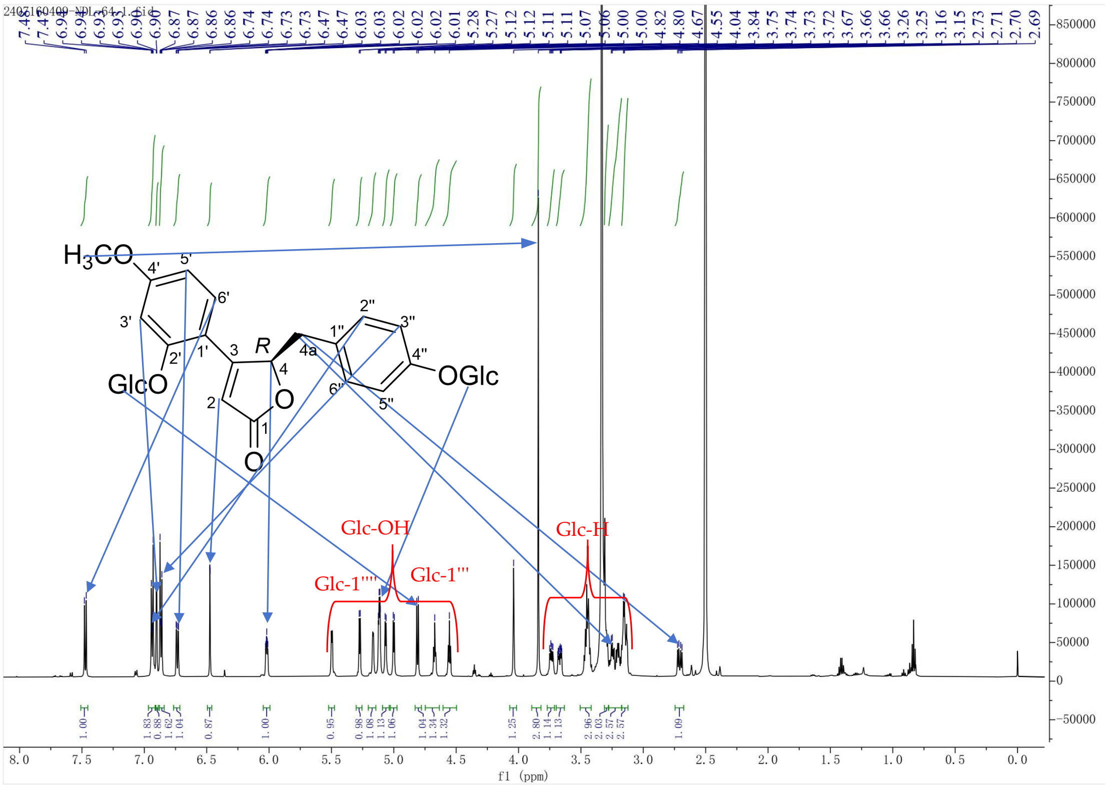

Figure S5 ^{1}H-NMR Spectrum of **1**

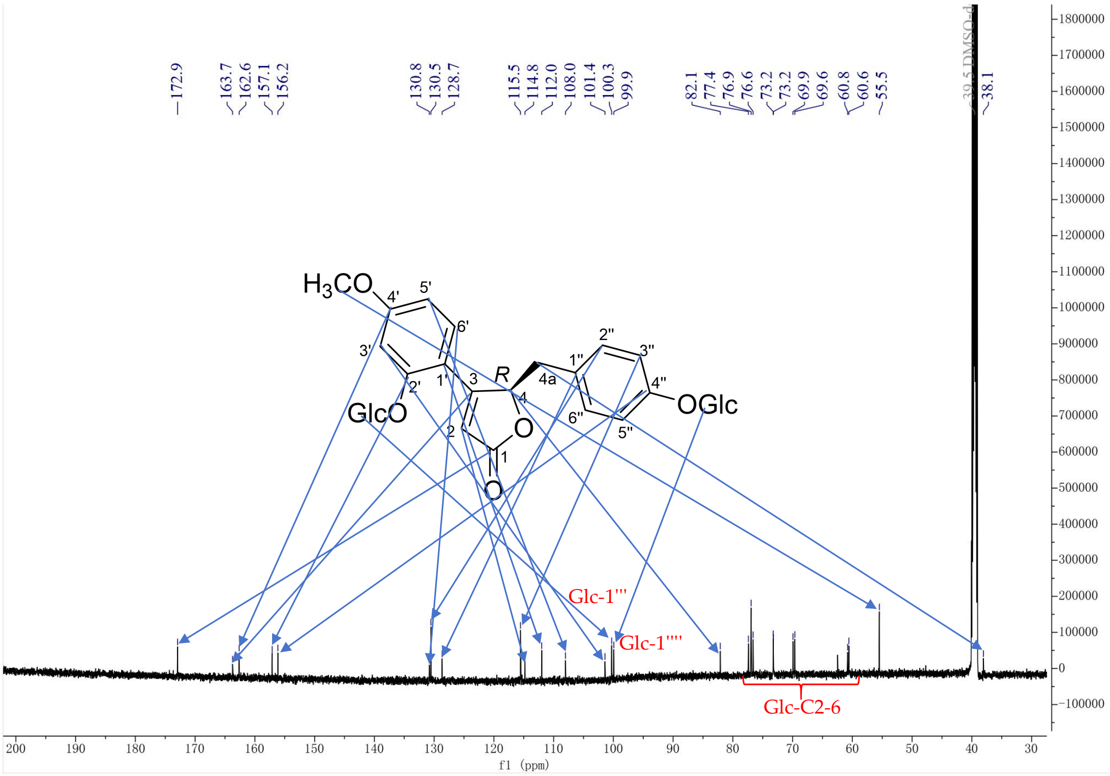

Figure S6 ^{13}C-NMR Spectrum of **1**

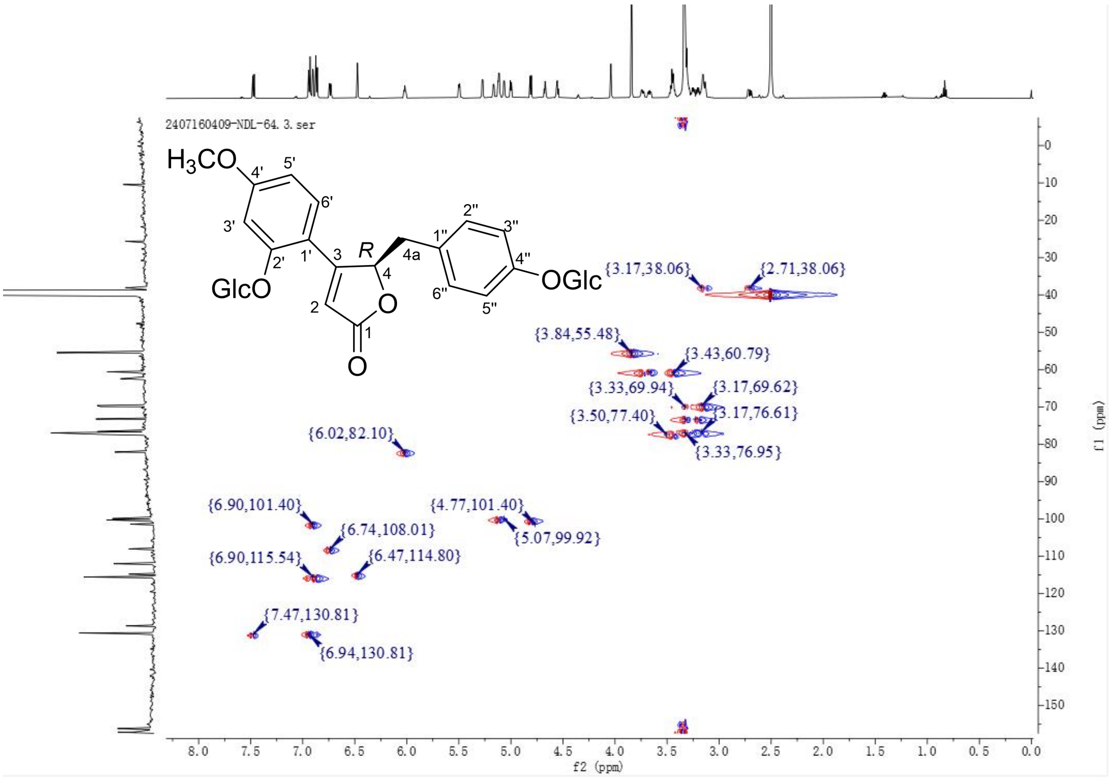

Figure S7 HSQC Spectrum of **1**

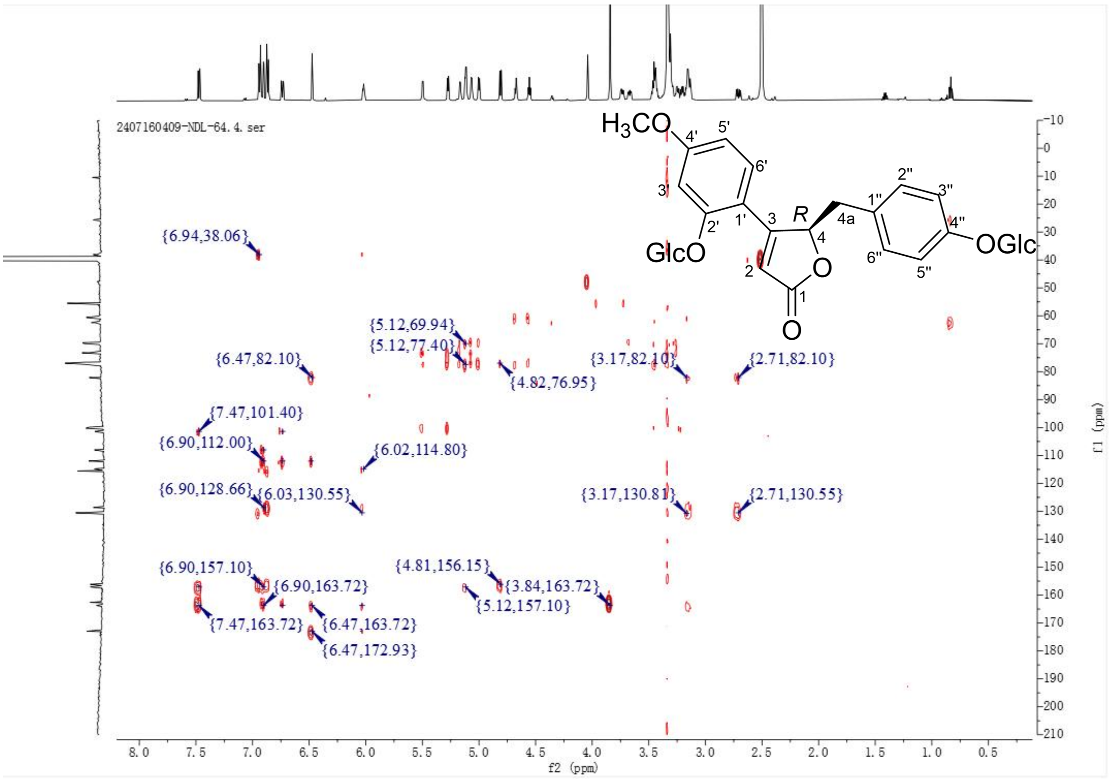

Figure S8 HMBC Spectrum of **1**

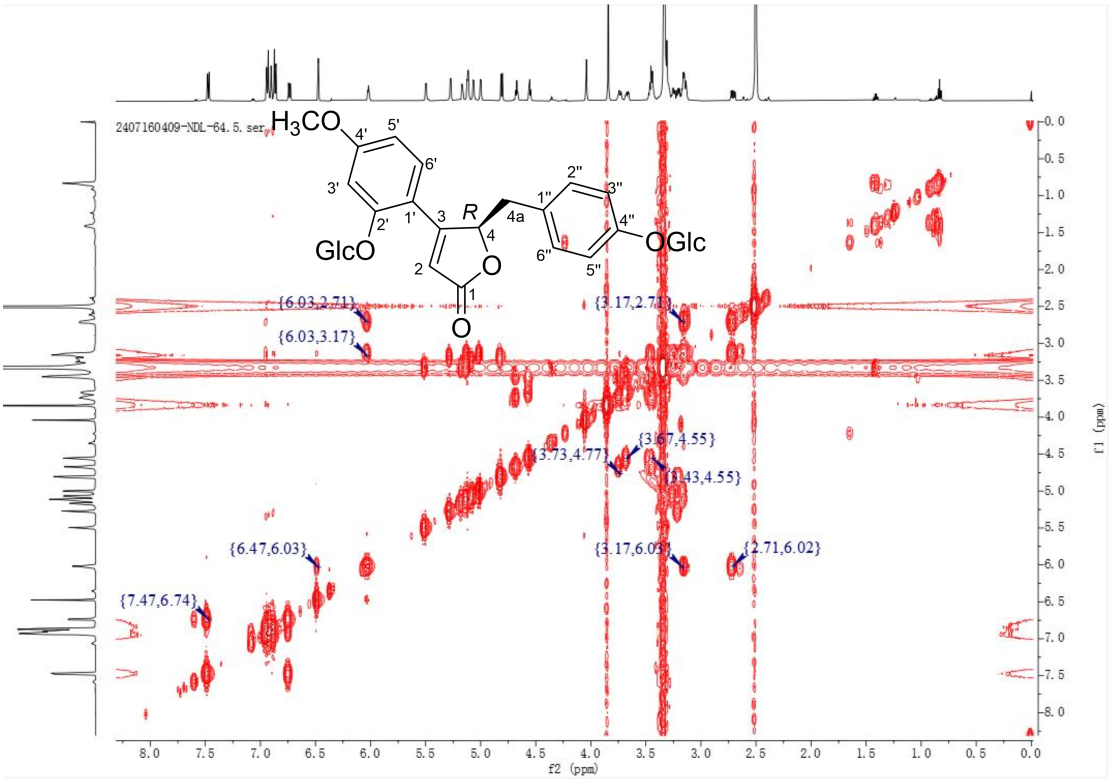

Figure S9 ^{1}H-^{1}H COSY Spectrum of **1**

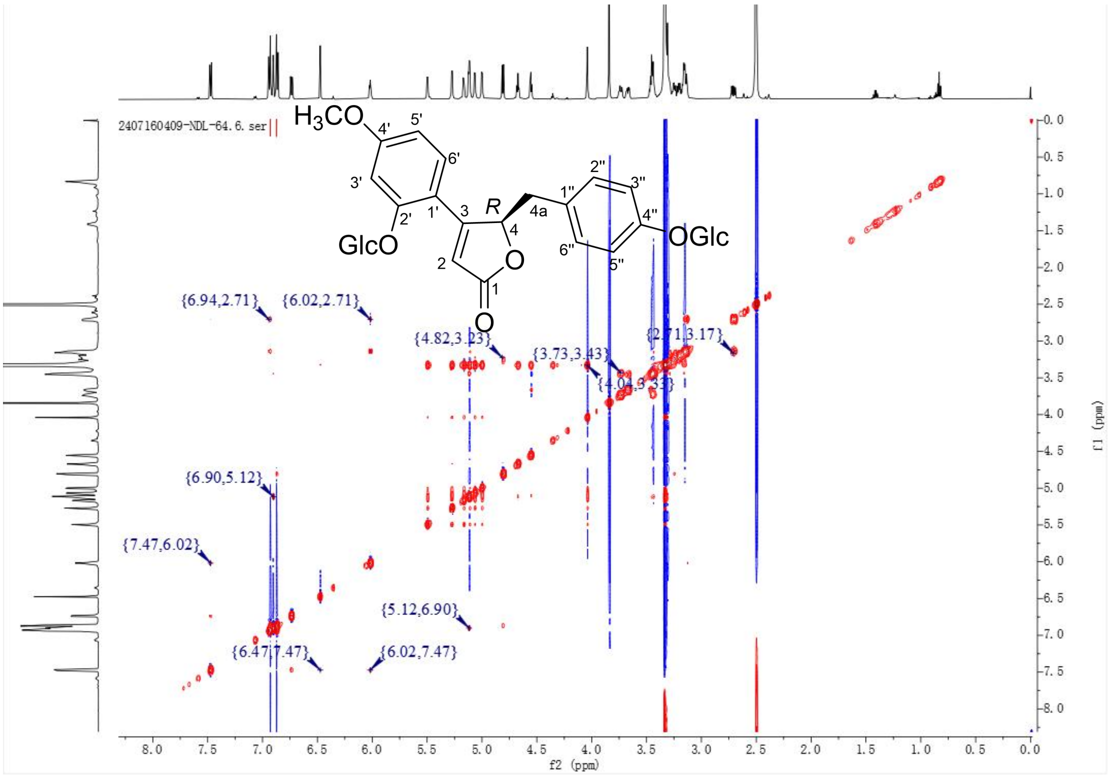

Figure S10 NOESY Spectrum of **1**

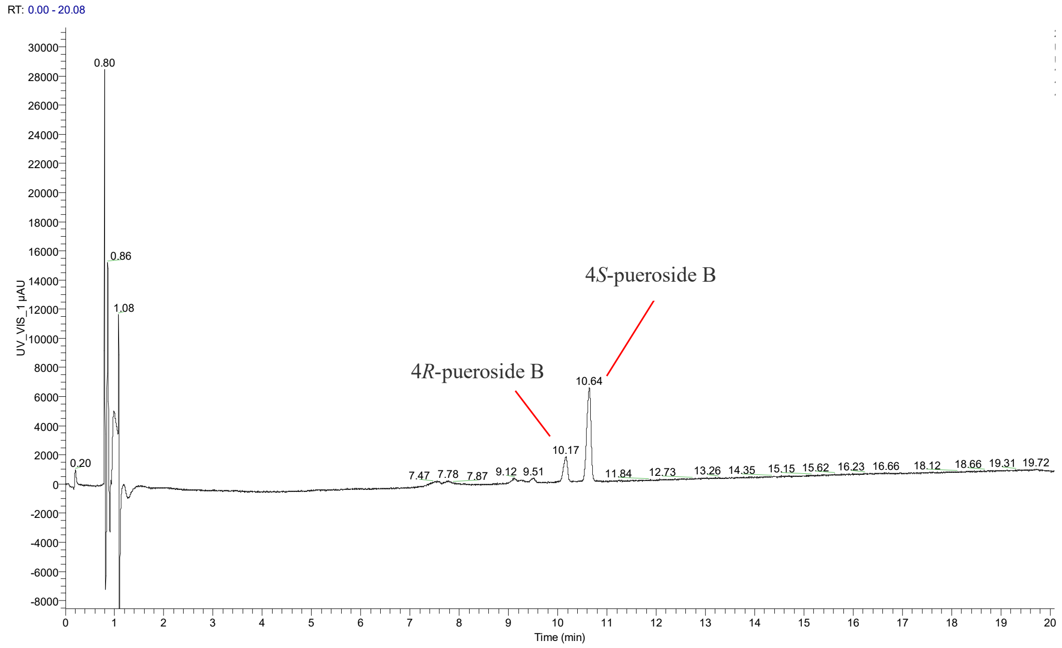

Figure S11 HPLC chromatogram of **1** (4*R*-pueroside) and **2** (4*S*-pueroside)

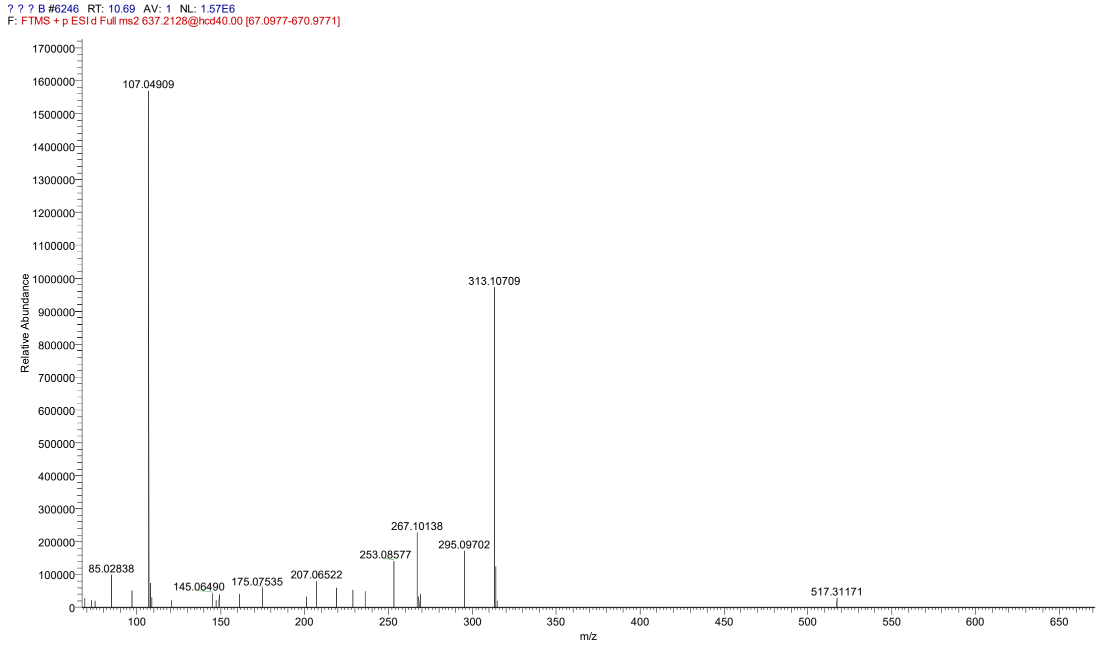

Figure S12 MS / MS fragment diagram of **1**

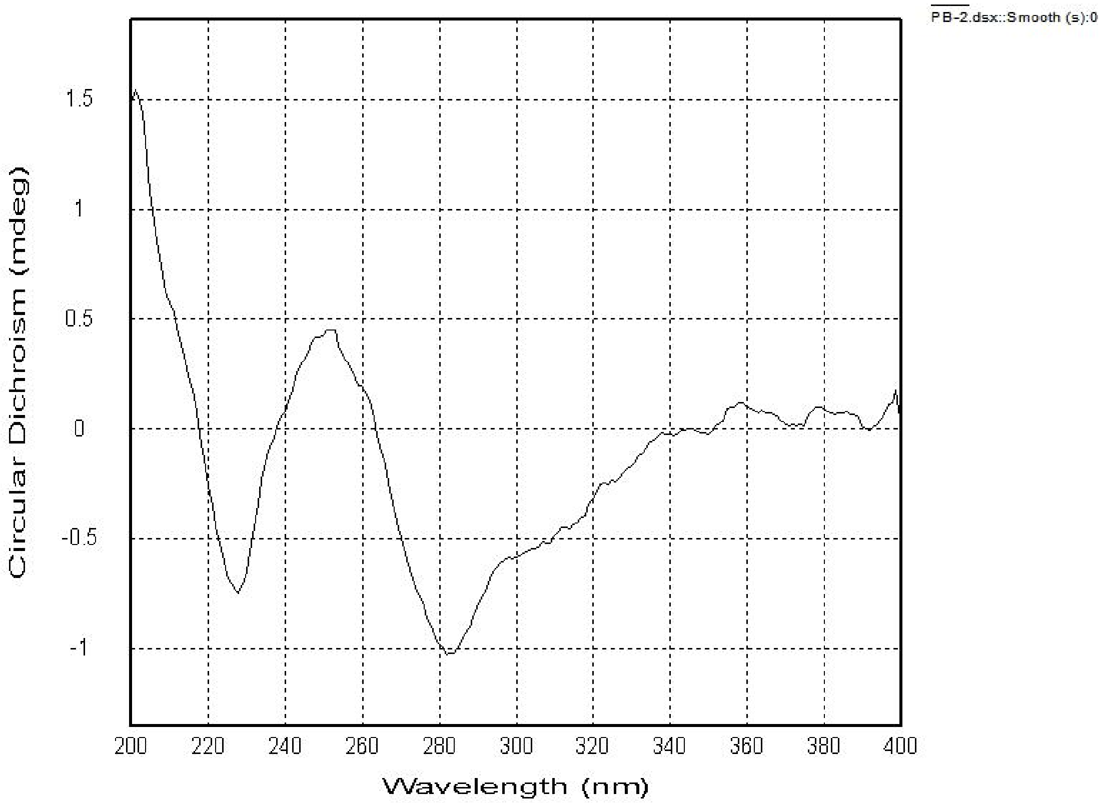

Figure S13 CD Spectrum of **2**

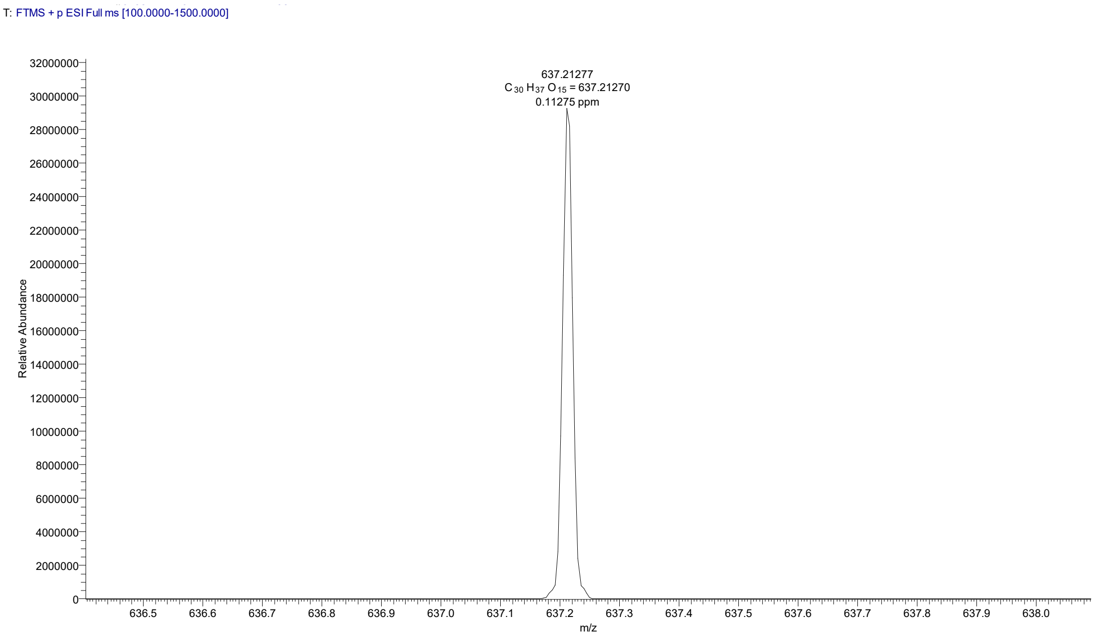

Figure S14 ESI-HR-MS Spectrum of **2**

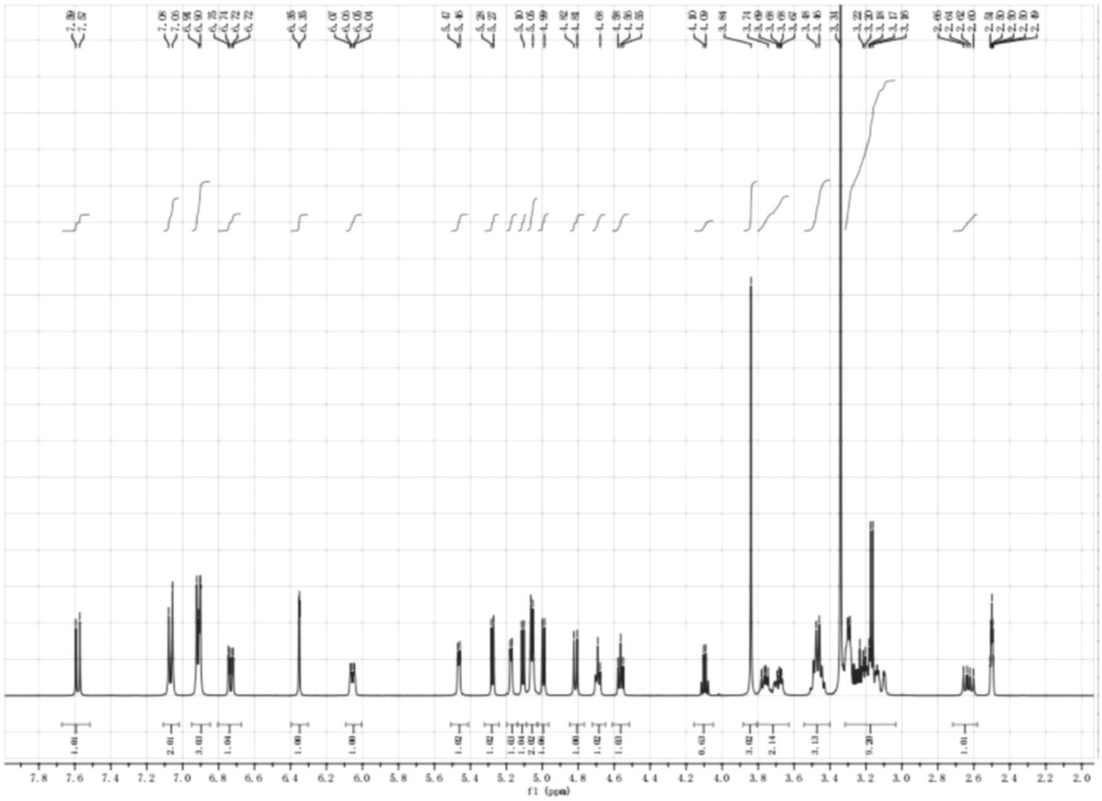

Figure S15 ^{1}H-NMR Spectrum of **2**

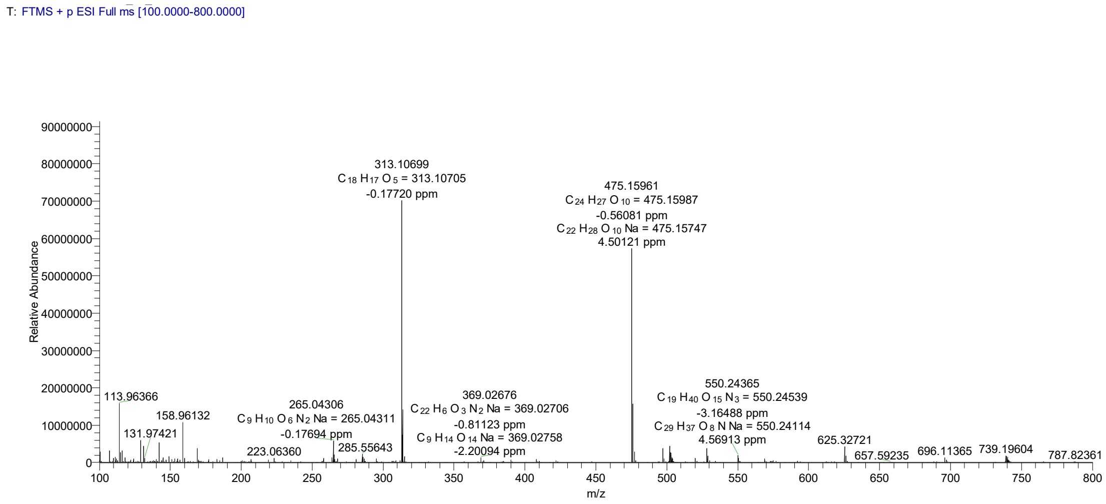

Figure S16 ESI-HR-MS Spectrum of **3**

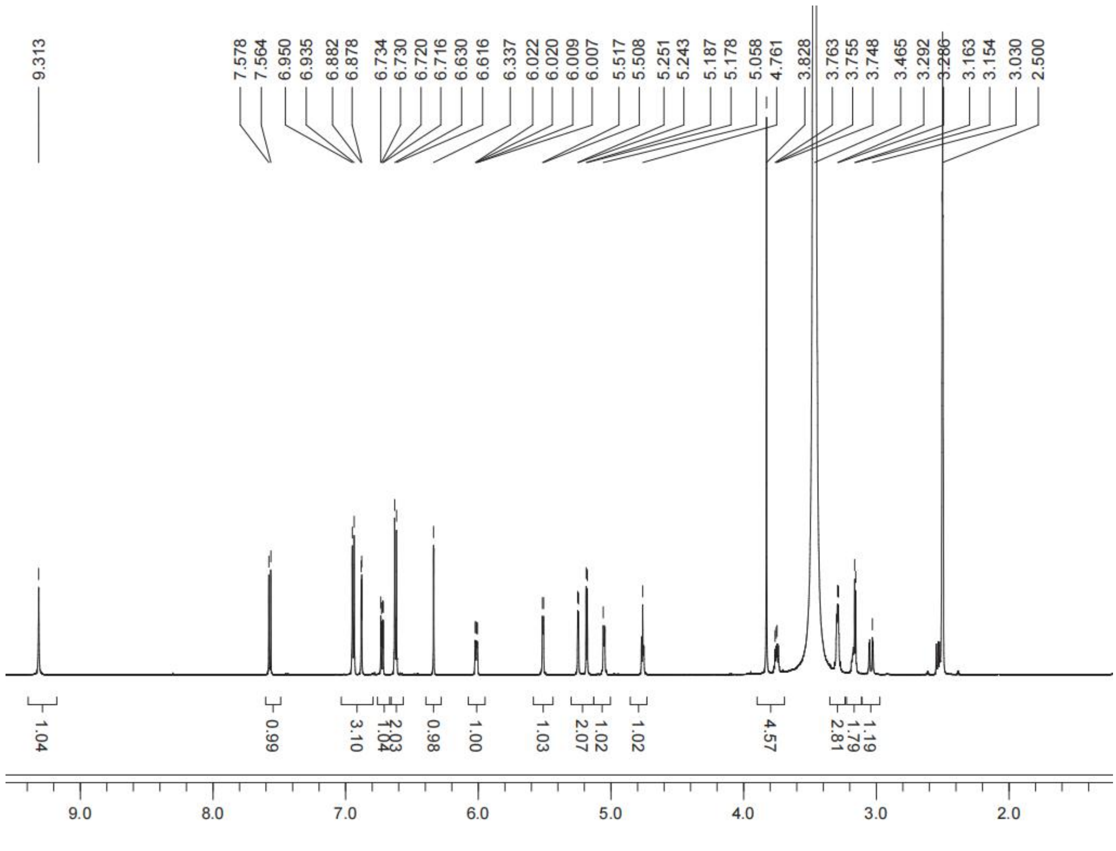

Figure S17 ^{1}H-NMR Spectrum of **3**

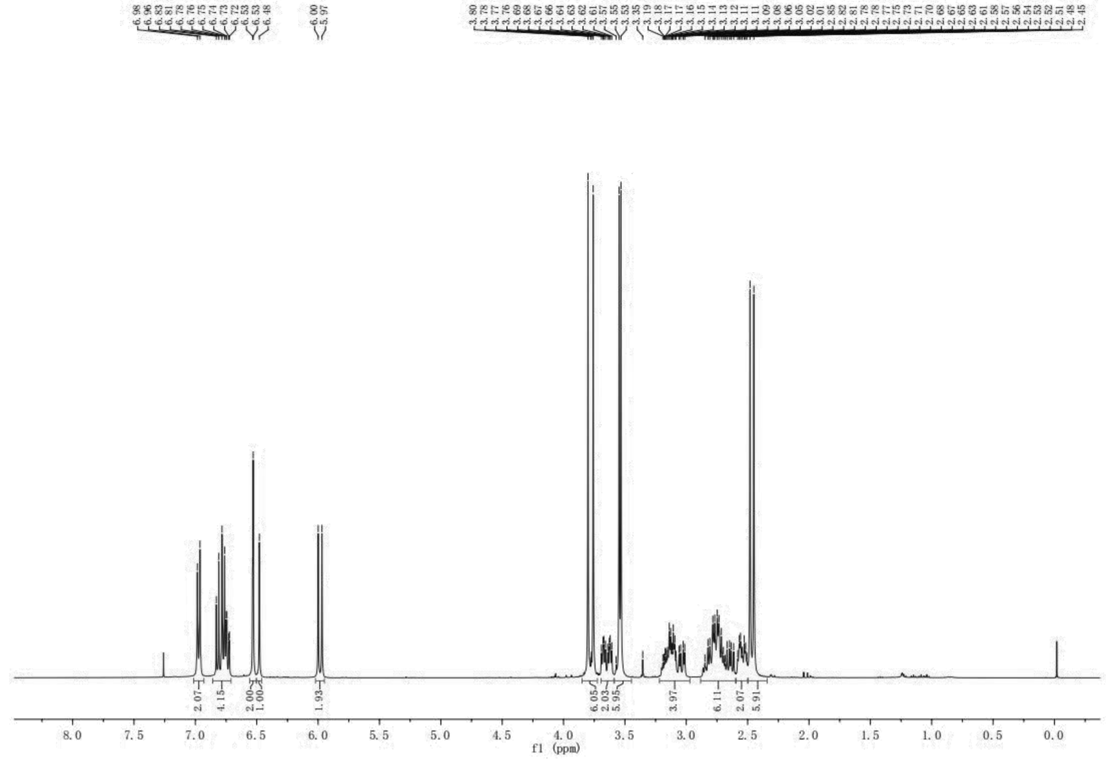

Figure S18 ^{1}H-NMR Spectrum of **4**

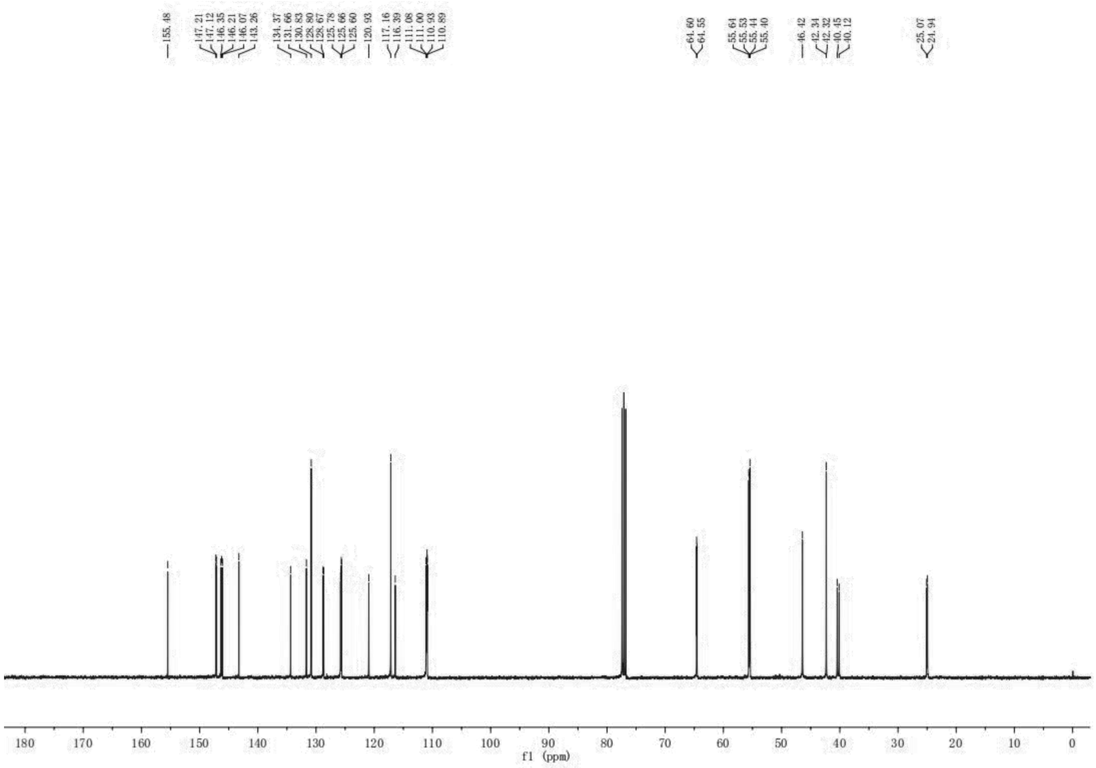

Figure S19 ^{13}C-NMR Spectrum of **4**

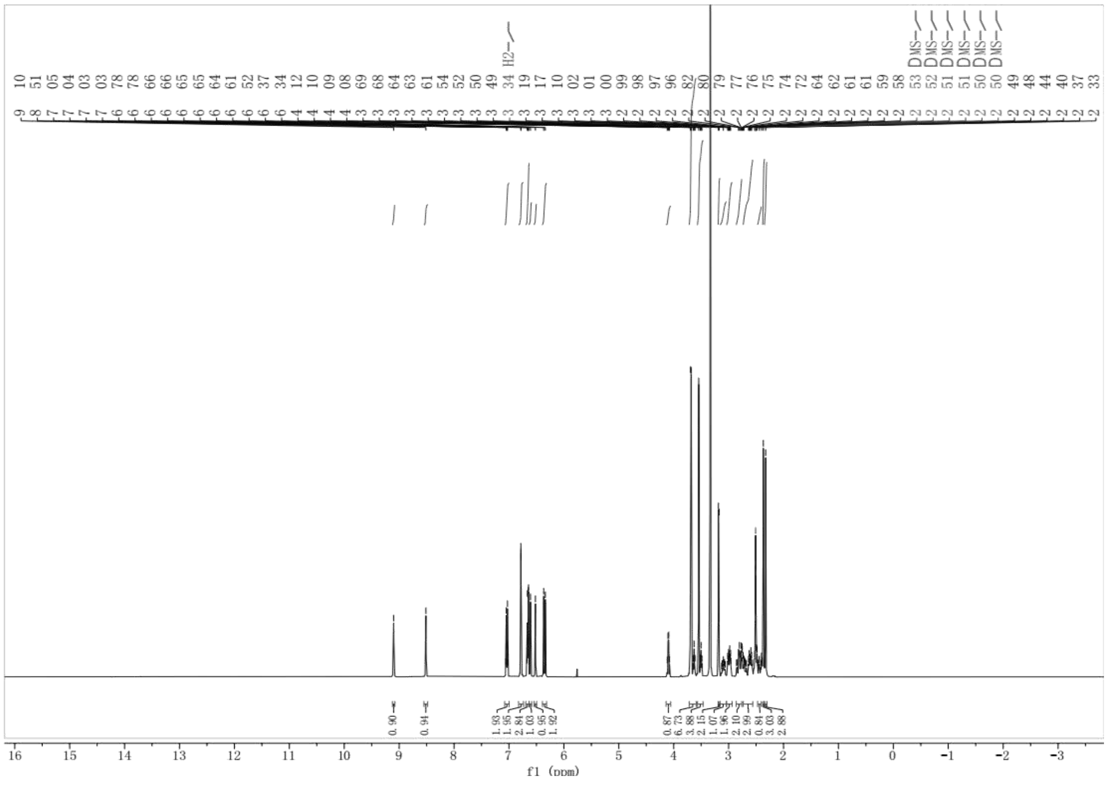

Figure S20 ^{1}H-NMR Spectrum of **5**

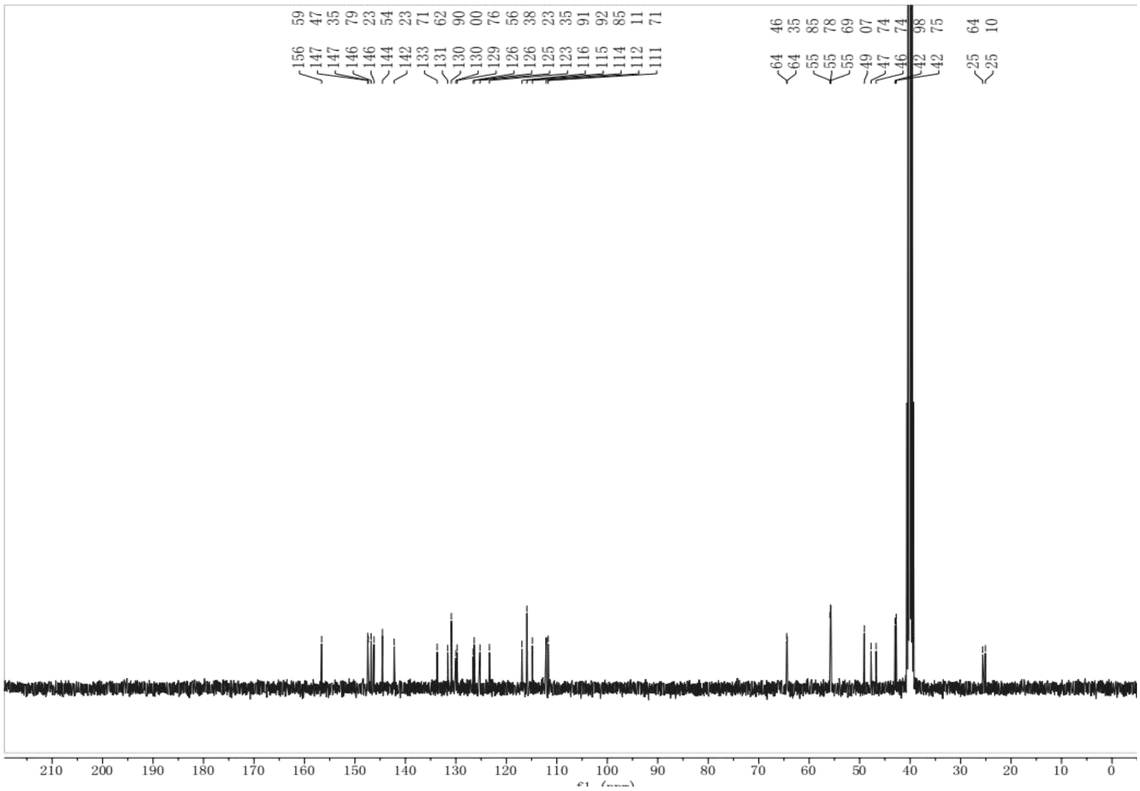

Figure S21 ^{13}C-NMR Spectrum of **5**

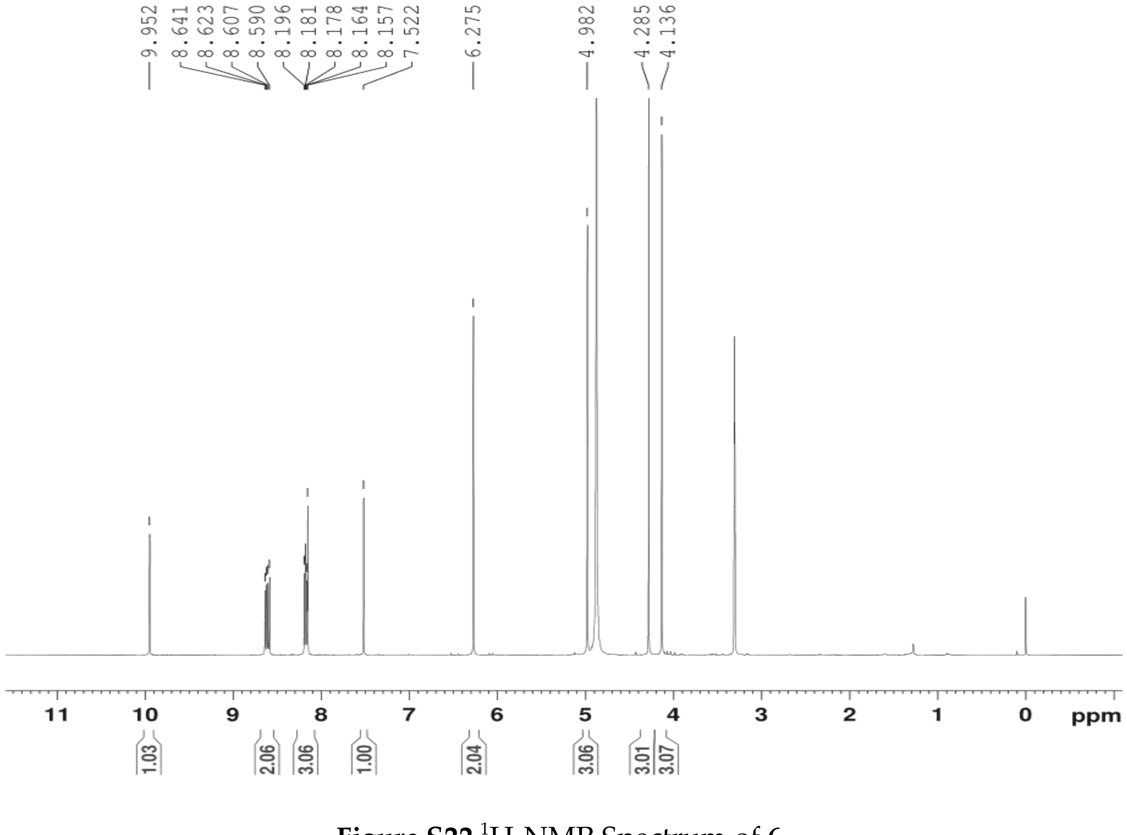

Figure S22 ^{1}H-NMR Spectrum of **6**

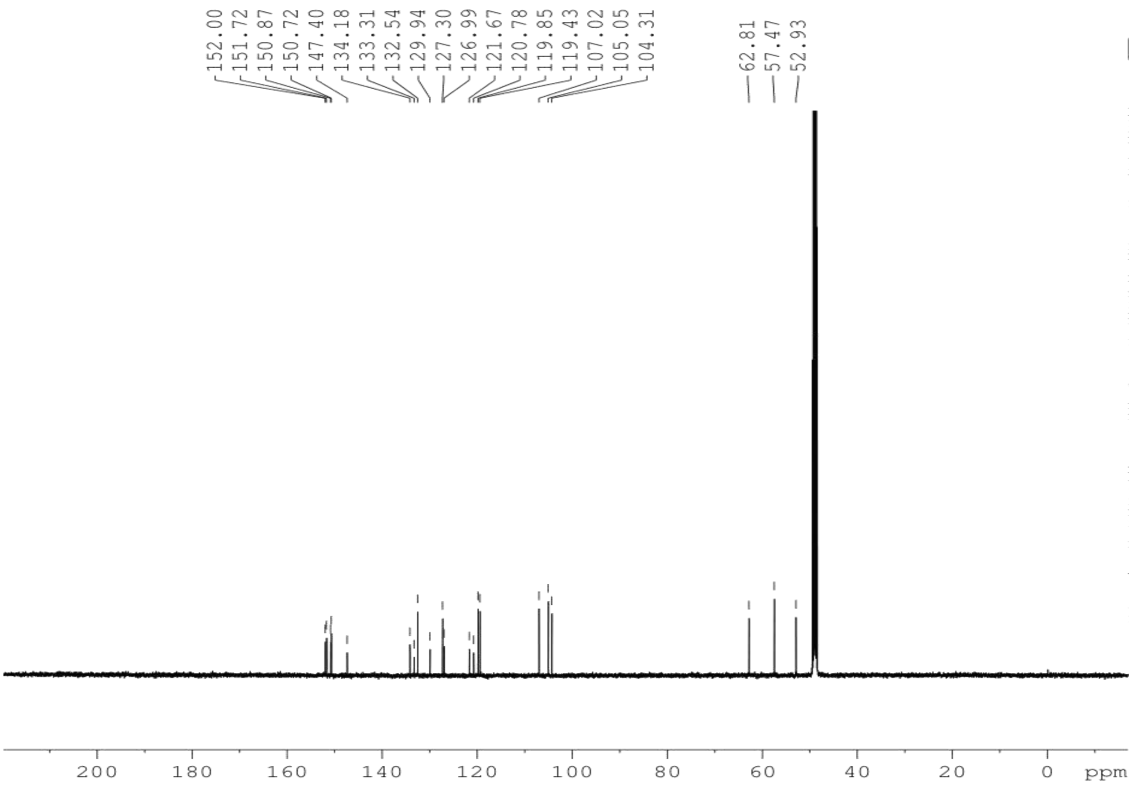

Figure S23 ^{13}C-NMR Spectrum of **6**

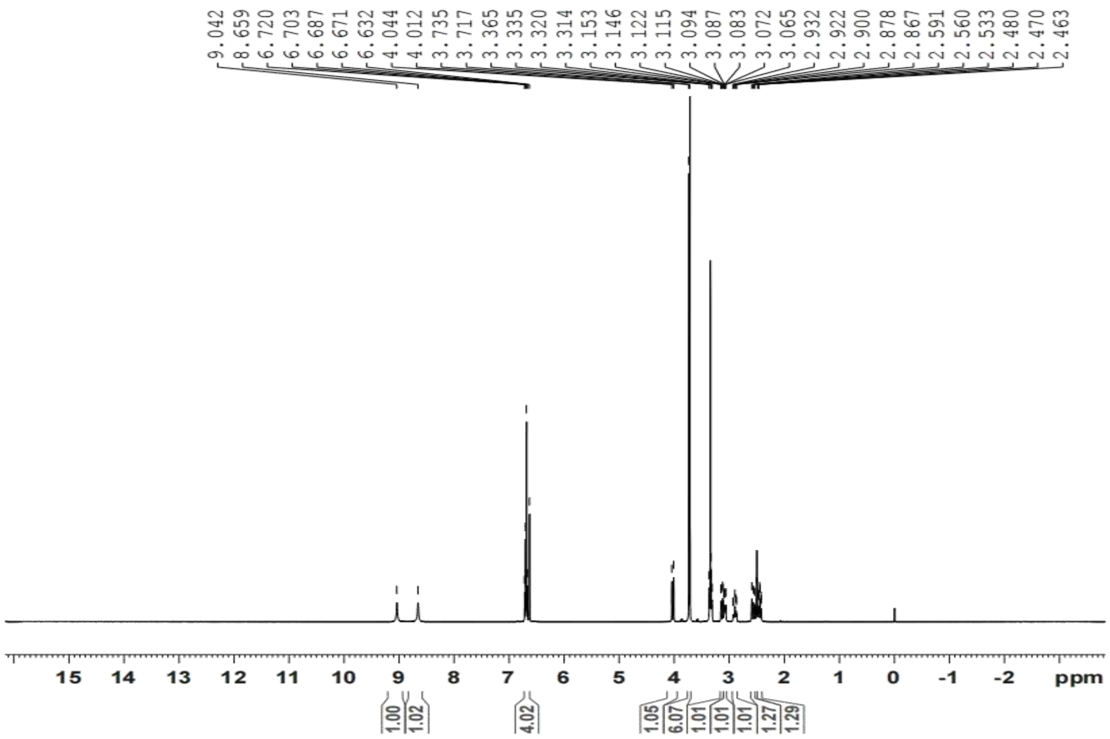

Figure S24 ^{1}H-NMR Spectrum of **7**

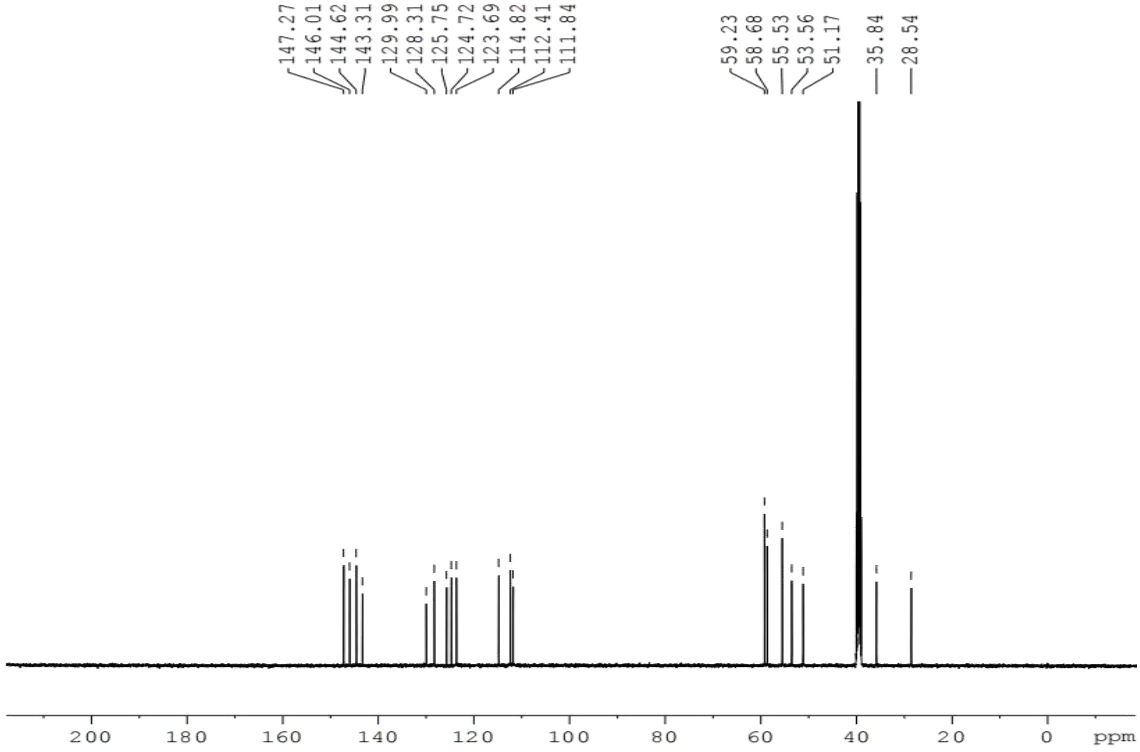

Figure S25 ^{13}C-NMR Spectrum of **7**

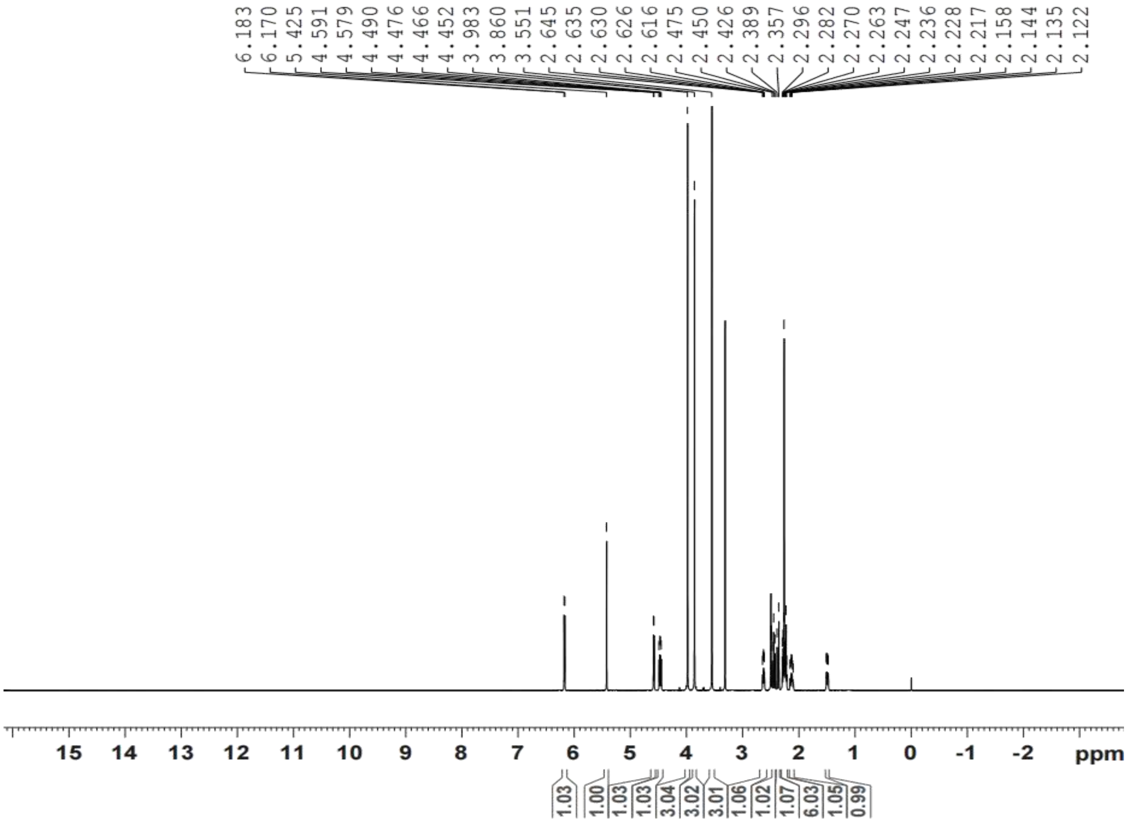

Figure S26 ^{1}H-NMR Spectrum of **8**

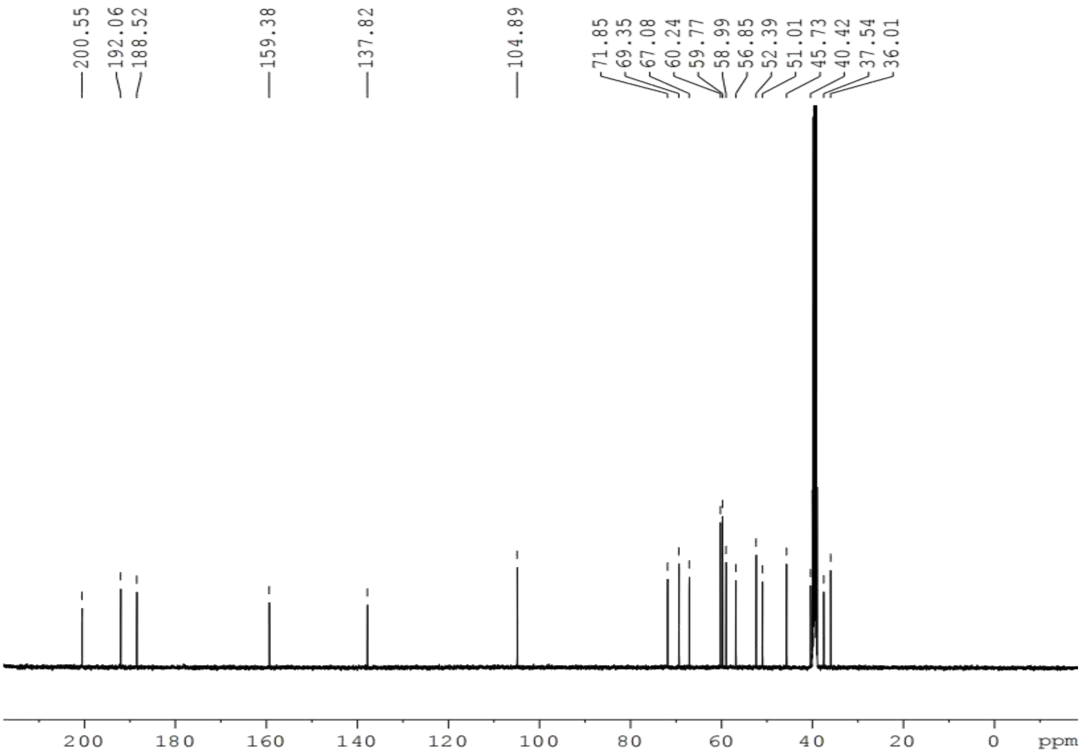

Figure S27 ^{13}C-NMR Spectrum of **8**

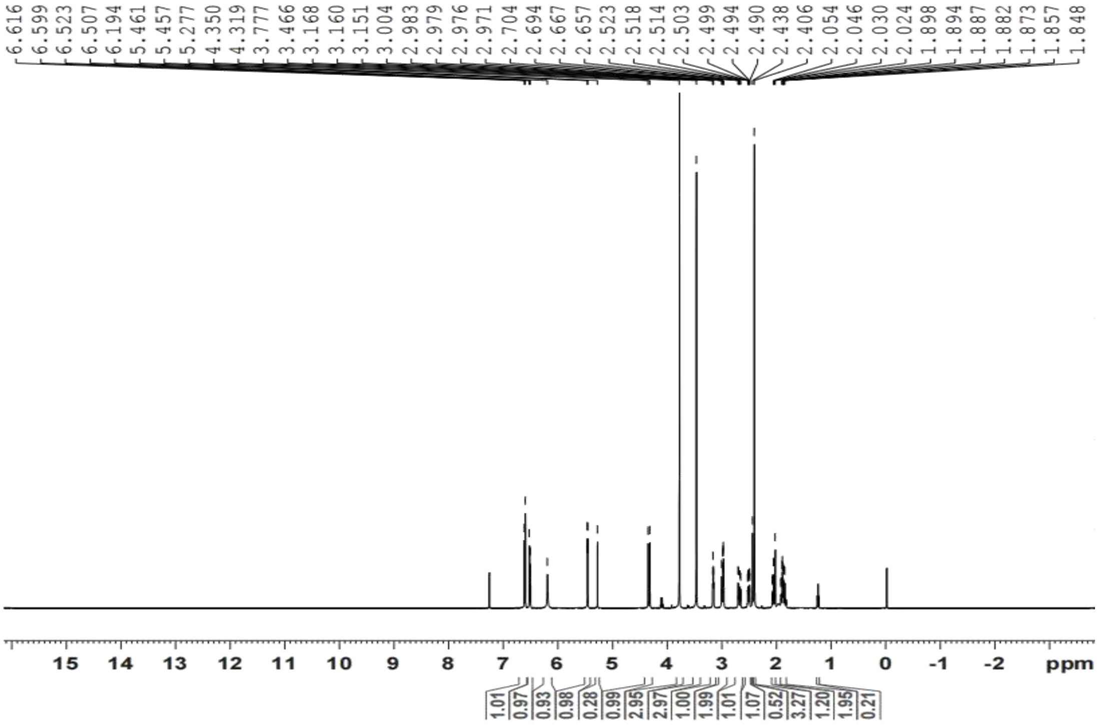

Figure S28 ^{1}H-NMR Spectrum of **9**

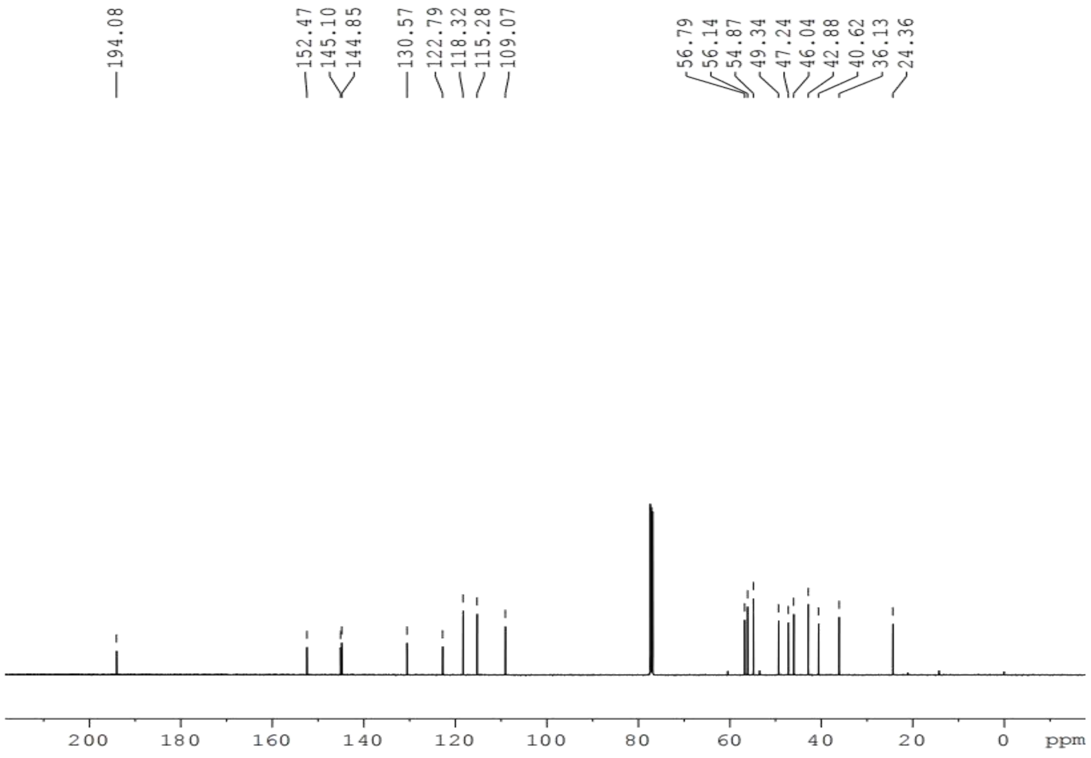

Figure S29 ^{13}C-NMR Spectrum of **9**

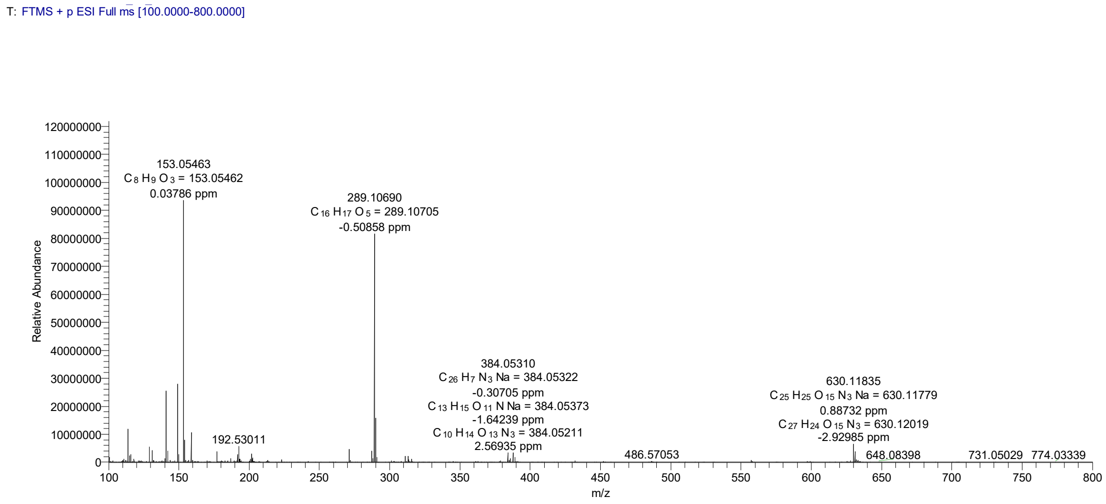

Figure S30 ESI-HR-MS Spectrum of **10**

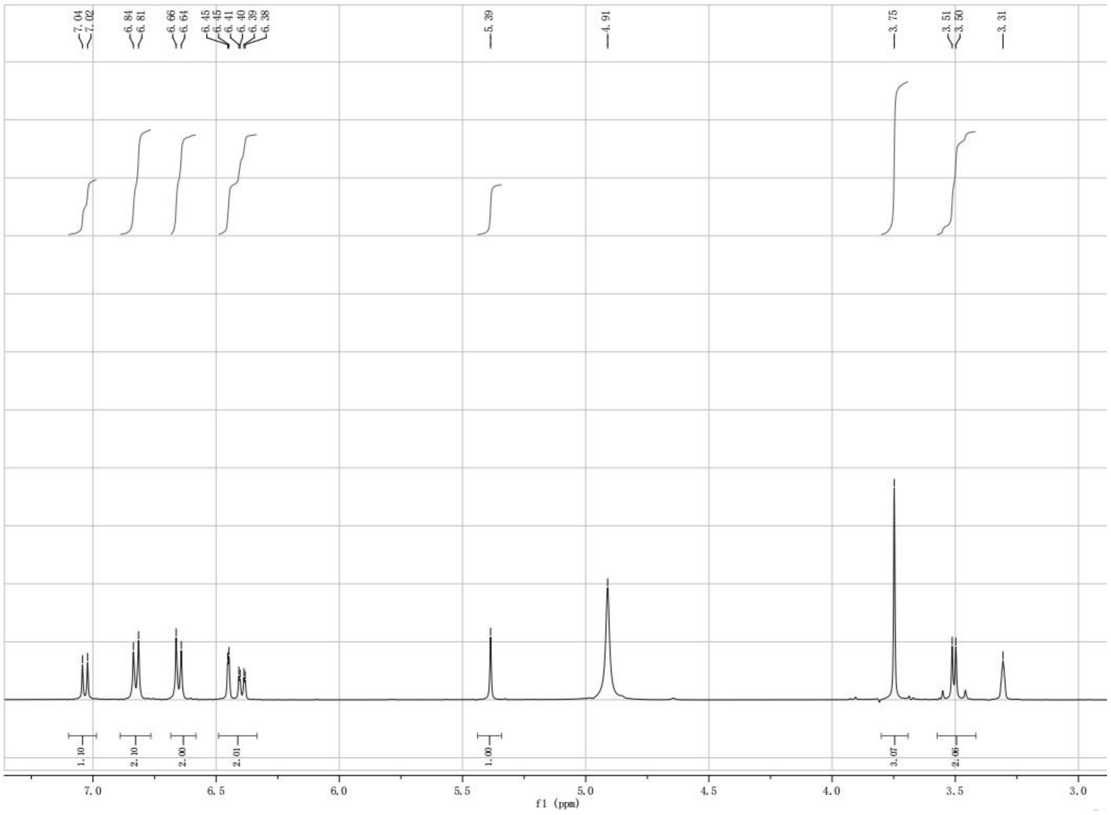

Figure S31 ^{1}H-NMR Spectrum of **10**

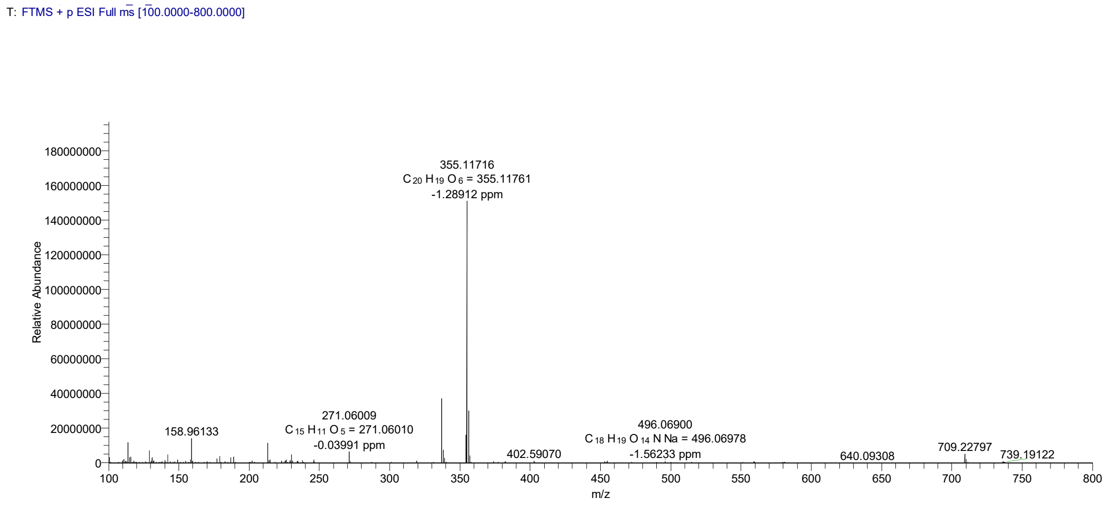

Figure S32 ESI-HR-MS Spectrum of **11**

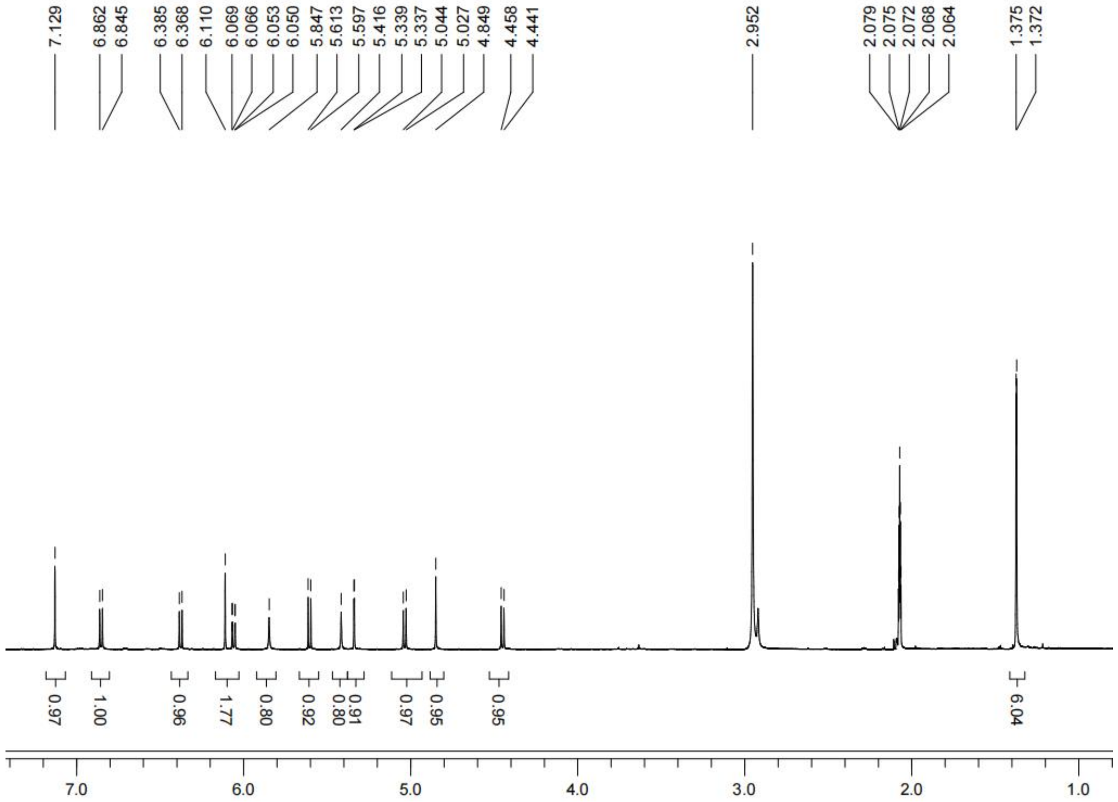

Figure S33 ^{1}H-NMR Spectrum of **11**

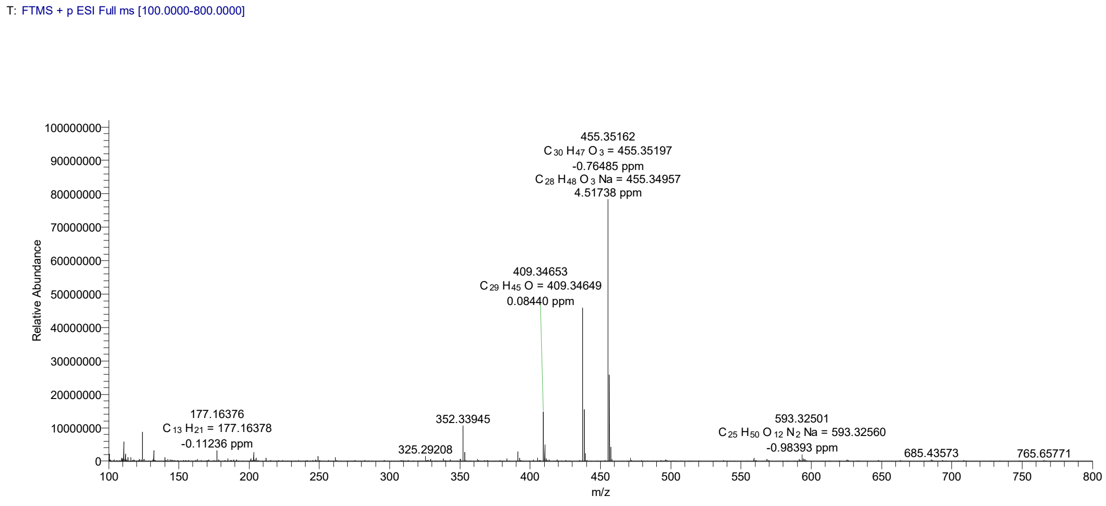

Figure S34 ESI-HR-MS Spectrum of **12**

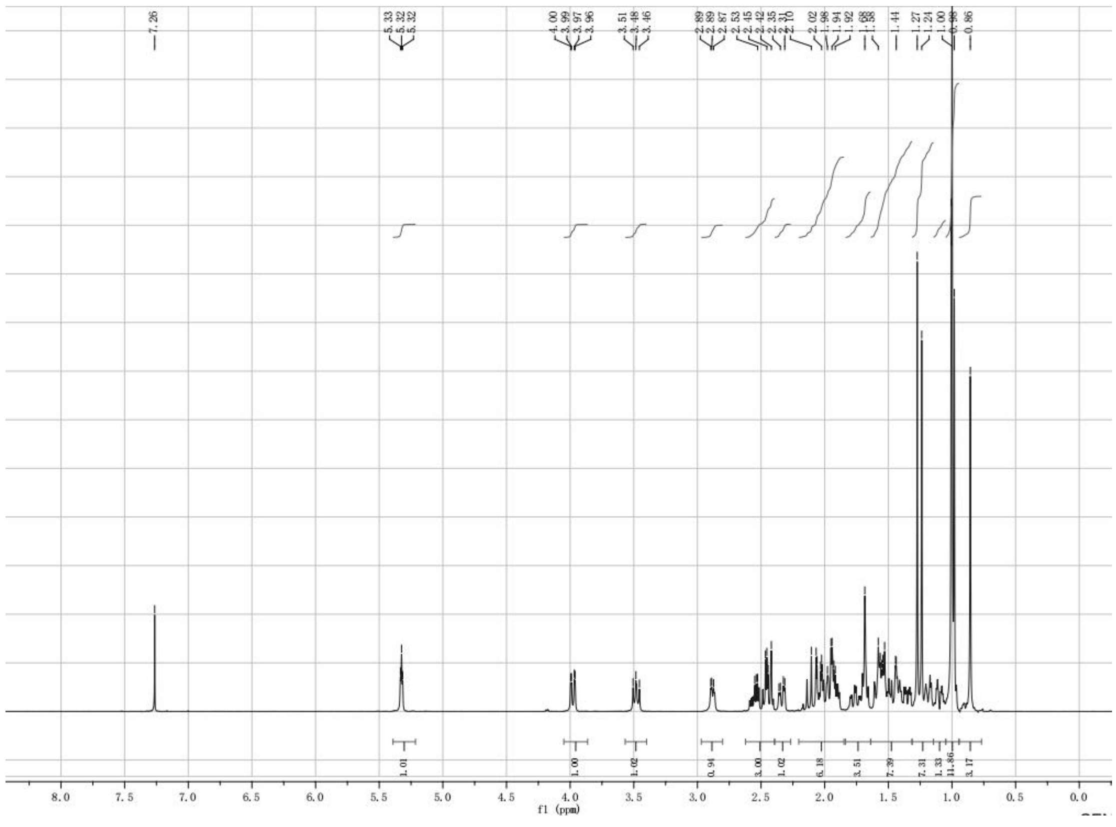

Figure S35 ^{1}H-NMR Spectrum of **12**
